# Comprehensive analysis of differentially expressed genes and transcriptional regulation induced by salt stress in two contrasting cotton genotypes

**DOI:** 10.1186/1471-2164-15-760

**Published:** 2014-09-05

**Authors:** Zhen Peng, Shoupu He, Wenfang Gong, Junling Sun, Zhaoe Pan, Feifei Xu, Yanli Lu, Xiongming Du

**Affiliations:** State Key Laboratory of Cotton Biology/Institute of Cotton Research, Chinese Academy of Agricultural Sciences, 455000 Anyang, Henan China; Maize Research Institute, Sichuan Agricultural University, 611130 Wenjiang, Sichuan China

**Keywords:** Cotton, Salt stress, Leaf transcriptome, Transcription factor, MicroRNA

## Abstract

**Background:**

Cotton (*Gossypium* spp.) is one of the major fibre crops of the world. Although it is classified as salt tolerant crop, cotton growth and productivity are adversely affected by high salinity, especially at germination and seedling stages. Identification of genes and miRNAs responsible for salt tolerance in upland cotton (*Gossypium hirsutum* L.) would help reveal the molecular mechanisms of salt tolerance. We performed physiological experiments and transcriptome sequencing (mRNA-seq and small RNA-seq) of cotton leaves under salt stress using Illumina sequencing technology.

**Results:**

We investigated two distinct salt stress phases—dehydration (4 h) and ionic stress (osmotic restoration; 24 h)—that were identified by physiological changes of 14-day-old seedlings of two cotton genotypes, one salt tolerant and the other salt sensitive, during a 72-h NaCl exposure. A comparative transcriptomics was used to monitor gene and miRNA differential expression at two time points (4 and 24 h) in leaves of the two cotton genotypes under salinity conditions. The expression patterns of differentially co-expressed unigenes were divided into six groups using short time-servies expression miner software. During a 24-h salt exposure, 819 transcription factor unigenes were differentially expressed in both genotypes, with 129 unigenes specifically expressed in the salt-tolerant genotype. Under salt stress, 108 conserved miRNAs from known families were differentially expressed at two time points in the salt-tolerant genotype. We further analyzed the predicted target genes of these miRNAs along with the transcriptome for each time point. Important expressed genes encoding membrane receptors, transporters, and pathways involved in biosynthesis and signal transduction of calcium-dependent protein kinase, mitogen-activated protein kinase, and hormones (abscisic acid and ethylene) were up-regulated. We also analyzed the salt stress response of some key miRNAs and their target genes and found that the expressions of five of nine target genes exhibited significant inverse correlations with their corresponding miRNAs. On the basis of these results, we constructed molecular regulatory pathways and a potential regulatory network for these salt-responsive miRNAs.

**Conclusions:**

Our comprehensive transcriptome analysis has provided new insights into salt-stress response of upland cotton. The results should contribute to the development of genetically modified cotton with salt tolerance.

**Electronic supplementary material:**

The online version of this article (doi:10.1186/1471-2164-15-760) contains supplementary material, which is available to authorized users.

## Background

High soil salinity, a devastating environmental stress, typically causes major reductions in crop productivity and quality [1]. Over 800 million hectares, equivalent to 6.5% of the world’s total land area, are currently estimated to be impacted by high salt concentration [2]. In China, approximately 100 million hectares distributed over 16 provinces have been affected by increased salinity [3]. To stabilize global crop production, the problem of salinity must be urgently addressed.

Salt stress generally induces a combination of dehydration/osmotic-related effects and damage as a consequence of excess sodium ions that greatly affect plant growth and crop production [4]. Osmotic stress and Na^+^ stress are considered to be the two major components of the plant salt-stress response [5]. Plants employ various mechanisms to deal with salt stress; these mechanisms include minimization of the amount of salt taken up by roots and its partitioning at tissue and cellular levels to avoid buildup of toxic concentrations in the cytosol of functional leaves [6, 7]. Much effort has been devoted to revealing the molecular mechanisms of plant salt tolerance, with the ultimate goal of improving salt tolerance of crop plants.

Cotton (*Gossypium hirsutum* L.) is not only the world’s leading textile fiber, but is also a major oil crop. Although cotton is the second most salt-tolerant herbaceous crop [8, 9], its growth and productivity are adversely affected by high salinity, especially at germination and the young seedling stage [10]. Salinity suppresses primary root growth [11], inhibits the length and numbers of secondary roots [12], and limits photosynthesis and respiration, flowering, boll and fiber quality, and ion uptake in cotton, resulting in significant yield losses [13]. Salt stress has also been found to regulate the expression level of many genes in different bio-processes and pathways, including morphological adaptation, maintenance of ion homeostasis, cell signal transduction, and oxidative stress mitigation [14–16]. Identifying salt-tolerance genes is an important component of the breeding of salt-tolerant crop plants through genetic engineering. Although many genes controlling response to high salinity have been identified in model plants, only a few salt stress-inducible genes, such as NHX1 (*GhNHX1*) [17], DREB (*GhDREB1*) [18], ERF (*GhERF2- GhERF6*) [19–21], NAC (*GhNAC1-GhNAC6*) [22], metallothionein (*GhMT3a*) [23], *GhMPK2* [24], *GhMKK1* [25], and CCCH-type zinc finger (*GhZFP1*) [26] have been documented in cotton. With recent advances in genomic sequencing and transcriptome mapping (microarray and high-throughput sequencing), some salt-related genes and regulatory factors have been identified in cotton on a large scale at the genome-wide level [27–33]. Nevertheless, the molecular basis of cotton tolerance to salt stress remains to be discovered.

Small RNAs are important post-transcriptional regulators of gene expression. miRNAs are a highly conserved class of endogenous, non-coding RNAs that range in length from 19 to 25 nucleotides (nt) [34]. In plants, cleavage or translational repression of target mRNA appears to be the prevalent method of post-transcriptional regulation [35, 36]. Current findings that some plant miRNAs respond to stress conditions and that some miRNA targets are stress-related genes suggest that miRNAs play important roles in plant stress response. miRNA expression profiles in response to salt stress have been analyzed in *Arabidopsis thaliana* [37], *Oryza sativa* [38], *Zea ma*ys [39], *Gossypium hirsutum* [40], and *Caragana intermedia* [41]. In addition to studying the effects of stress on miRNAs, the identification of miRNA targets is important. Among cotton cultivars, however, some miRNAs related to salt tolerance have been found to differ [42–45]. Little is known regarding their expression profiles in response to salt stress, and their roles in salt adaptation remain unclear.

**Figure 1:**
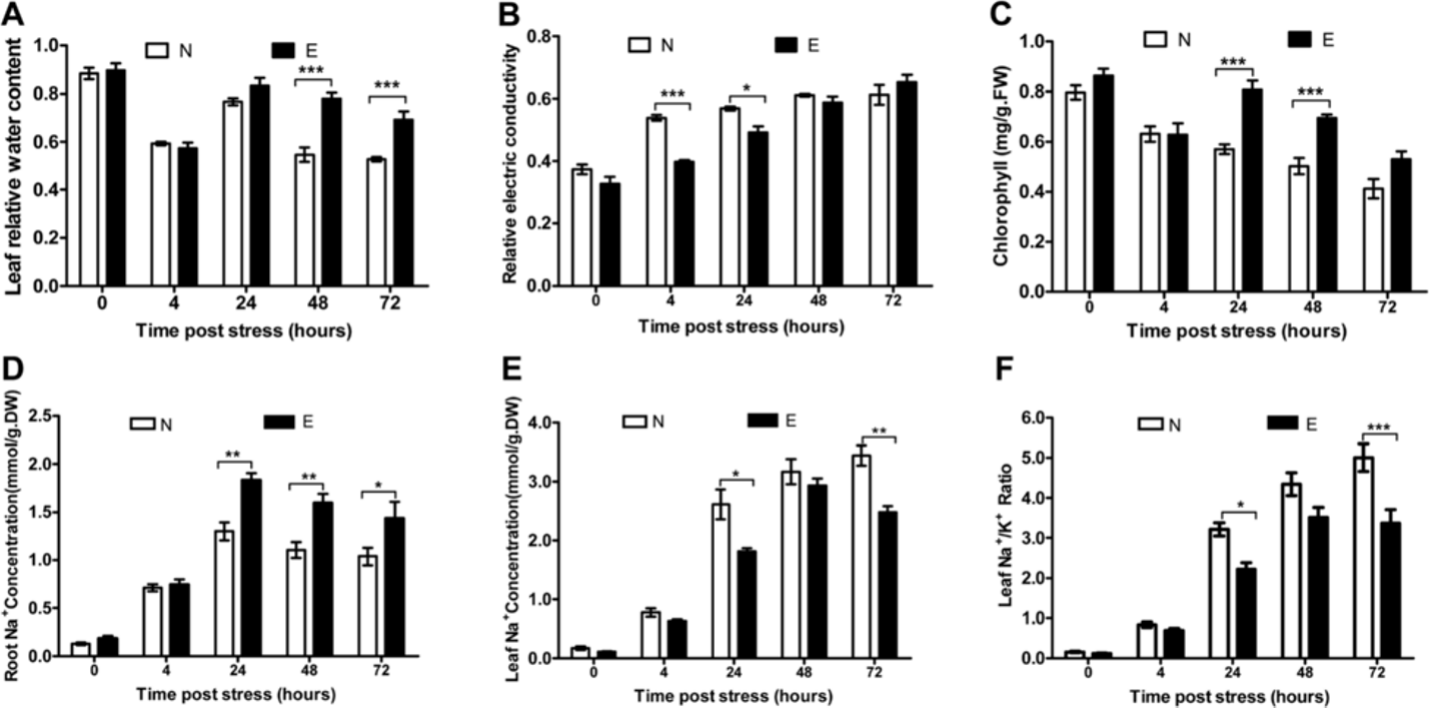
**Physiological analysis of Earlistaple 7 and Nan Dan Ba Di Da Hua in response to various durations of salt stress. (A)** Leaf relative water content (RWC) levels; **(B)** Leaf relative electrical conductivity (REC); **(C)** Leaf chlorophyll-a and chlorophyll-b content; **(D)** Root Na^+^ concentration; **(E)** Leaf Na^+^ concentration; **(F)** Ratio of leaf K^+^/Na^+^ concentration in 200 mM NaCl-treated and non-treated samples. Data represent means ± SE of three independent experiments (*n* = 3 or 9); * *P* < 0.05; ** *P* < 0.01; *** *P* < 0.001.

Transcriptome (mRNA-seq and small RNA-seq) analysis has recently emerged as a powerful approach to excavate large numbers of genes and miRNA sequences [45, 46]. Although some differentially expressed salt-related genes have been identified by this technique after short- or long-term salt treatment, comparative transcriptional changes in response to salt stress between early and late periods have not been reported. An additional limitation is that most reported gene expression profiles related to salt resistance have been derived from a single genotype [47–49]. In those studies, it was consequently difficult to accurately distinguish true salt tolerance-related genes from salt-responsive ones. Furthermore, most research for salt stress focused on root [31, 46]. However, the previous results show that when subjected to salt stress in upland cotton, mostly absorbed Na^+^ accumulated in the shoot through the roots, and the root of K^+^ transport to the leaves to maintain high K^+^/ Na^+^ in the leaves [9]. Otherwise, in our study, Na^+^ concentration in leaf also showed significant difference between two lines (Figure 1E), lower Na^+^ concentration could reduce the damage of leaf photosynthesis, further improved the salt tolerance. These results indicated that the shoot, especially the leaves of salt tolerant cotton must have some special mechanisms to alleviate Na^+^ toxicity under salt stress, therefore, we chose the leaf as object of study. Thus, comparative analyses of gene and miRNA expression in seedling leaves during two stages of salt stress may further elucidate molecular mechanisms of salt tolerance in cotton.

To obtain insights into the initial transcriptional regulation and differential expression of functional genes in response to high salinity, we exposed two cotton genotypes (salt-tolerant Earlistaple 7 and salt-sensitive Nan Dan Ba Di Da Hua) to salt stress for 0–72 h. We then compared dynamic changes in ion accumulation and osmotic adjustment between Earlistaple 7 and Nan Dan Ba Di Da Hua and analyzed the expression profiles of responsive genes and miRNAs. According to their expression patterns, salt stress-inducible genes could be broadly discerned as early-, late-, and sustained-induced regulated genes, and their functional significance was characterized. We also identified and characterized conserved miRNAs in the two genotypes at two time points (4 and 24 h) under salt stress. Putative target genes of these miRNAs were predicted from the transcripts by computational methods. We established a model of the miRNA-mediated regulatory network by combining the mRNA-seq and small RNA-seq information. Our comprehensive physiological- and molecular-level analysis of salt stress responses should facilitate illumination of the mechanisms of salt stress tolerance in *G. hirsutum*.

## Results

### Physiological differences between salt-sensitive and salt-tolerant genotypes under salinity stress

Genotypes Nan Dan Ba Di Da Hua and Earlistaple 7 were subjected to short-term salinity stress (200 mM NaCl) at the seedling stage for 4, 24, 48, and 72-h. Seedling response to salt was evaluated based on leaf relative water content (RWC), relative electrical conductivity (REC), and chlorophyll and ion (Na^+^ and K^+^) content of leaves and roots (Figure 1).

Highly significant (*P* < 0.001) differences were observed in leaf RWC and chlorophyll content between the two genotypes after 48- and 24-h salt treatments, respectively, although these values were sharply decreased in both genotypes after 4 h of exposure to NaCl compared with controls (Figure 1A, C). After 4 and 24 h of treatment, the REC of Nan Dan Ba Di Da Hua was highly significantly (*P* < 0.001) increased throughout the duration of stress, while that of Earlistaple 7 was maintained at a lower level. After 48 h, however, the RECs of the two genotypes were similar (Figure 1B). As shown in Figure 1D–F, leaf and root Na^+^ concentrations increased in both genotypes under salinity stress. Characteristically, Nan Dan Ba Di Da Hua roots accrued less Na^+^ as compared with Earlistaple 7 (Figure 1D), whereas Earlistaple 7 contained a significantly lower level of leaf Na^+^ (Figure 1E, F). This difference can probably be attributed to the higher Na^+^ absorption capacity of roots of salt-tolerant Earlistaple 7 compared with salt-sensitive Nan Dan Ba Di Da Hua, which prevents the transport of excessive Na^+^ to the seedling shoots.

Visual damage owing to salinity stress appeared on the leaves of Nan Dan Ba Di Da Hua beginning approximately 24 to 72 h after 200-mM NaCl treatment. After 0.5 h, distinct wilting and dehydration caused by osmotic stress [50] was observed on leaves of both genotypes. By 4 h, both genotypes showed more severe wilting, which was followed by gradual recovery to an upright position (24 h). The restoration of Earlistaple 7 was obviously better than that of Nan Dan Ba Di Da Hua, which experienced slight wilting of cotyledons and old leaves, and dry necrosis of leaf edges. After 48 h, these phenomena were markedly more noticeable in both genotypes, although plants of Earlistaple 7 were in much better condition than those of Nan Dan Ba Di Da Hua. According to the above results, significant physiological and morphological differences were observed at different salt-treatment time points in response to salt stress, which was accordingly divided into two typical phases: dehydration stress (4 h) and ionic stress (24 h). We therefore selected these two time points for further study.

### *De novo*mRNA-seq assembly and annotation of non-redundant unigenes

Six cDNA and six small RNA libraries were constructed from total RNA extracted from 14-day-old seedling leaves of two upland cottons (cultivars Nan Dan Ba Di Da Hua and Earlistaple 7) treated with 200 mM NaCl (or water as a control) for 4 and 24 h.

Sample data from the six different libraries are summarized in Table 1. After quality control, approximately 169,850,000 valid reads and roughly 7.9 Gb of nucleotides were obtained. The overall data retention rate was high (average of 82.72%), and the data quality was acceptable. Because the whole-genome sequence of upland cotton is currently not publically available, the valid reads from the six libraries were merged for *de novo* assembly (Table 1). After removal of repeats from the spliced sequences, 415,429 transcripts with lengths ≥200 bp were obtained. The total length of all transcripts was approximately 236 Mb. The longest transcript for each locus was taken as the unigene, resulting in 143,080 unigenes comprising about 54 Mb of nucleotides (Table 2). The length of these assembled unigenes ranged from 200 to 2,000 bp. The overall length distribution and GC content of the spliced unigenes are presented in Additional file 1.

**Table 1:** **The data quality of mRNA-seq and the transcripts in two genotypes**

Summary		Nan Dan Ba Di Da Hua			Earlistaple 7		
		CK	4 h	24 h	CK	4 h	24 h
Raw data	Read	19,277,194	12,479,678	12,479,678	17,969,500	17,969,500	21,602,254
Raw data	Base	1,927,719,400	1,247,967,800	1,247,967,800	1,796,950,000	1,796,950,000	2,160,225,400
Valid data	Read	16,097034	10,492,532	10,492,532	15,026,640	15,026,640	18,100,020
Valid data	Base	1,497,064,827	977,315,799	977,315,799	1,396,615,990	1,396,615,990	1,685,347,764
Valid data	Average length	93	93.14	93.14	92.94	92.94	93.11
Valid data	Valid ratio	83.50%	84.08%	84.08%	83.62%	83.62%	83.79%
MapData	Number	13,688,457	12,721,295	8,976,783	10,560,871	12,677,667	15,465,674
MapData	Data%	85.04%	84.29%	85.55%	84.58%	84.37%	85.45%
ExpTranscript	Number	374,204	372,440	358,476	367,228	374,490	381,094
ExpTranscript	Sum.	809418.58	803094.61	785100.61	819293.67	805543.43	792193.63
ExpTranscript	Transcript%	87.97%	87.56%	84.28%	86.33%	88.04%	89.59%

**Table 2:** **Length distribution of the transcripts and unigenes clustered from the de novo assembly**

Category	Transcript	Unigene
All (> = 200 bp)	415,429	143,080
> = 500 bp	273,632	56,929
> = 1000 bp	181,131	30,038
N50	1,747	1,239
N90	497	277
Total length	236,414,577	53,791,217
Max length	14,995	14,995
Min length	201	201
Average length	1111.61	707.9

Note: The N50 size is computed by sorting all transcripts from largest to smallest and by determining the minimum set of transcripts whose sizes total 50% of the entire transcript and unigene was the same; N90 was counted in the similar way.

Functional annotation of transcripts was mainly based on BLAST (Blastx tools) homology searches against various public protein databases (Table 3). Of the 143,080 non-redundant unigenes, 60,714 (42.43%) showed a significant similarity to known proteins in the NR database and 33,992 (23.76%) had significant hits in the SWISS-PROT database. These results suggested an abundance of newly discovered unigenes. Gene Ontology (GO) analysis classified most of the 143,080 annotated unigenes into GO functional categories of biological process, cellular component, and molecular function (see Additional file 1).

**Table 3:** **The numbers and distribution rate of unigenes in the databases of NR, SWISS-PROT, TrEMBL, CDD, PRAM, KOG and KEEG**

Unigene no.	NR	SWISS-PROT	TrEMBL	CDD	PFAM	KOG	KEGG
143080	60,714	33,992	62,473	29,641	54,865	17,958	20,241
	42.43%	23.76%	43.660%	20.72%	38.35%	12.55%	14.15%

Note: Comparison of the all unigenes with public databases like Non-redundant nucleotide database (NR), SWISS-PROT ((UniProt), TrEMBL and PFAM, KOG and KEGG, respectively, functional annotation was done through gene similarity >30% and e-value <1e^-5^.

### Identification of salt-responsive, differentially expressed unigenes

To identify genes displaying significant expression changes during NaCl treatment, differentially expressed unigenes (DEUs) were analyzed by comparing 4- and 24-h libraries with the control library for both Nan Dan Ba Di Da Hua and Earlistaple 7 (Table 4). Salt-sensitive and salt-tolerant genotypes showed very similar expression patterns. As the salt treatment duration lengthened, an increasing number of gene expression changes were observed in both genotypes. In addition, the number of up-regulated genes in the salt-tolerant genotype (Earlistaple 7) was more than in salt-sensitive genotype.

**Table 4:** **The numbers of all differentially expressed unigenes (DEUs) at different salt-stressed time points of two genotypes**

Items	Nan Dan Ba Di Da Hua		Earlistaple 7	
	0 versus 4 h	0 versus 24 h	0 versus 4 h	0 versus 24 h
Total	66,803	71,267	62,928	71,311
Up-regulated	32,789(49%)	31,423(44%)	33,790(54%)	40,131(56%)
Down-regulated	34,014(51%)	39,844(56%)	29,138(46%)	31,180(44%)

Note: Number of DEUs (*P* < 0.05 and |log_2_Ratio| ≥ 1) under NaCl stress for 4-h and 24-h as compared to their respective control samples.

**Figure 2:**
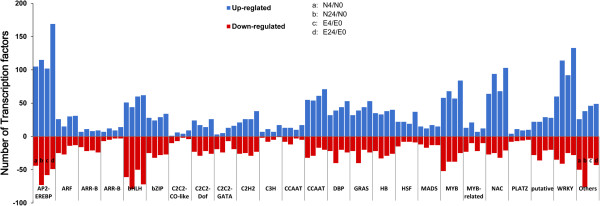
**Families of differentially expressed transcription factor unigenes from four comparisons.** The *x-axis* indicates the distribution of different transcription factor families in the four comparisons, and the *y-axis* represents the number of differentially expressed (up- or down-regulated) unigenes in each transcription factor family. The four comparisons are N4/N0, N24/N0, E4/E0, and E24/E0, where N and E indicate Nan Dan Ba Di Da Hua and Earlistaple 7, respectively, treated with 200 mM NaCl for either 4 or 24 h compared with the non-treated control (0 h). Further information is presented in Additional file 2B.

Transcription factors (TFs) play key roles in modulating the acclimation response of plants to severe environments. In our study, 2.2% (3,172) of the 143,080 unigenes encoding TF family members in Earlistaple 7 and Nan Dan Ba Di Da Hua were responsive to 4- and 24-h treatments with 200 mM NaCl. These unigenes were classified into 52 TF families and 1 group of putative TFs (see Additional file 2), including several key regulatory gene families involved in response to abiotic and biotic stress, such as AP2-EREBP (311), ARF (106), bHLH (254), bZIP (104), C2C2-Dof (90), C2H2 (94), DBP (163), GRAS (130), HB (136), MYB (225), NAC (170), and WRKY (222). Among these TFs, AP2-EREBP, MYB, NAC, and WRKY members unigenes were mostly up-regulated under salt stress, while bHLH and C2C2 families were represented by a balanced number of up- and down-regulated members (see Additional file 2). Many identified TFs belonged to AP2/EREBP, and most were involved in stress response. These results imply that AP2/EREBP family members play an important role in regulation of salt stress tolerance in *G. hirsutum*. MYB, NAC, and WRKY were also highly enriched during 4- and 24-h salt stress (Figure 2).

### Dynamic change analysis of co-expressed unigenes

To understand dynamic expression patterns of DEUs under salt stress at different time points, the various gene expression modes in Earlistaple 7 were identified. The most representative significantly DEU expression mode was then selected for further analysis. To uncover the mechanisms underlying salt sensitivity in Nan Dan Ba Di Da Hua, we used Short Time-series Expression Miner (STEM) software to analyze abundance changes of differentially expressed TF unigenes and other significant functional unigenes filtered by different genotypes at different time points. The analytical procedure used is outlined in Additional file 3.

**Figure 3:**
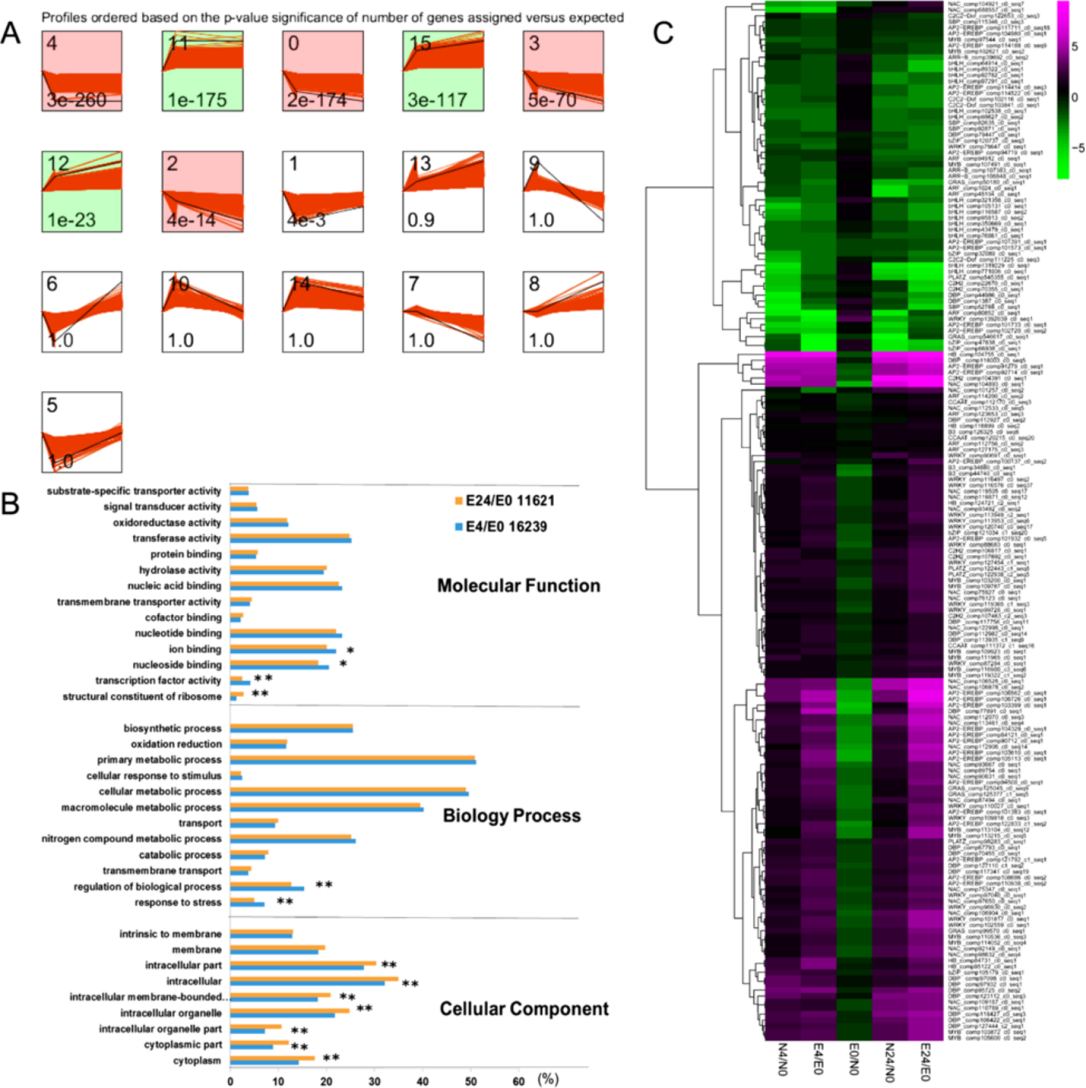
**Dynamic change analysis of differentially co-expressed unigenes. (A)** Dynamic expression pattern profiles after NaCl stress treatment. STEM clustering analysis was performed to identify clusters; each cluster contained various numbers of genes with similar expression patterns under NaCl stress. The top left hand corner indicates the ID of the cluster. The lower left hand corner contains the *P-*value of the number of assigned genes compared with the expected value. The black lines show model expression profiles. The red lines represent all individual gene expression profiles. The *x-axis* represents the stress treatment time in hours. The time series was log-normalized to start at 0. The *y-axis* of all genes in a cluster box are at the same scale; **(B)** Gene Ontology categories assigned to differentially expressed unigenes (DEUs) in response to 200 mM NaCl stress for 4 and 24 h. The *x-axis* represents the percentage of unigene numbers, and the *y-axis* shows the GO subcategories (at a level of 3 or below in the GO hierarchy); * *P* < 0.05; ** *P* < 0.01; **(C)** Comparison of salt-responsive transcription factor unigene co-expressed in NaCl-stressed Nan Dan Ba Di Da Hua and Earlistaple 7 leaves from the two clusters using hierarchical cluster analysis. The log_2_ Ratio values of salt responsive DEUs were used for hierarchical cluster analysis with the R *pheatmap* package. Unigene expression values are scaled ranging from +5 (magenta) to -5 (green). Red represents up-regulated unigenes, green represents down-regulated unigenes, and black indicates no significant difference in unigene expression. Details of annotated unigenes shown on the right are provided in Additional file 2I.

The functional enrichment of genes with distinct patterns (among co-expressed gene clusters) in Earlistaple 7 was also analyzed using STEM software. Seven significant expression profiles (*P* < 0.001) were identified. As shown in Figure 3A, significantly different profiles were represented by different background colors (red or green). According to the background color, the seven most representative expression patterns were divided into two clusters, one containing four and one containing three profiles. Unigenes within the same cluster were considered to be co-expressed genes following the same expression pattern. Differentially expressed unigenes of Earlistaple 7 at two time points (4 and 24 h) were selected from the two clusters and subjected to GO category gene enrichment analysis. The two unigene set comparisons (Earlistaple 7 at 4 vs. 0 h [E4/E0] and Earlistaple 7 at 24 vs. 0 h [E24/E0]) that were enriched (*P* < 0.01) for certain GO categories (at a level of 3 or below in the GO hierarchy) are shown in Figure 3B. The 18 most abundant GO terms into which E4/E0 and E24/E0 unigene sets were distributed included many abiotic stress-related categories, such as “transferase activity”, “nucleic acid binding”, “nucleotide binding”, and “ion binding”. In addition, several GO terms showed significant differences in enrichment between 4- and 24-h salt treatments, with “Response to stress”, “regulation of biological process”, “transcription factor activity”, and “structural constituent of ribosome” most prominent. It is increasingly clear that stress and metabolic signaling networks interact, and that this interaction is important in plant response to abiotic stresses [51]. The remaining clusters were not enriched for GO categories because of the many unannotated genes in the transcriptome, suggesting that numerous pathways involved in stress tolerance are yet to be revealed.

**Figure 4:**
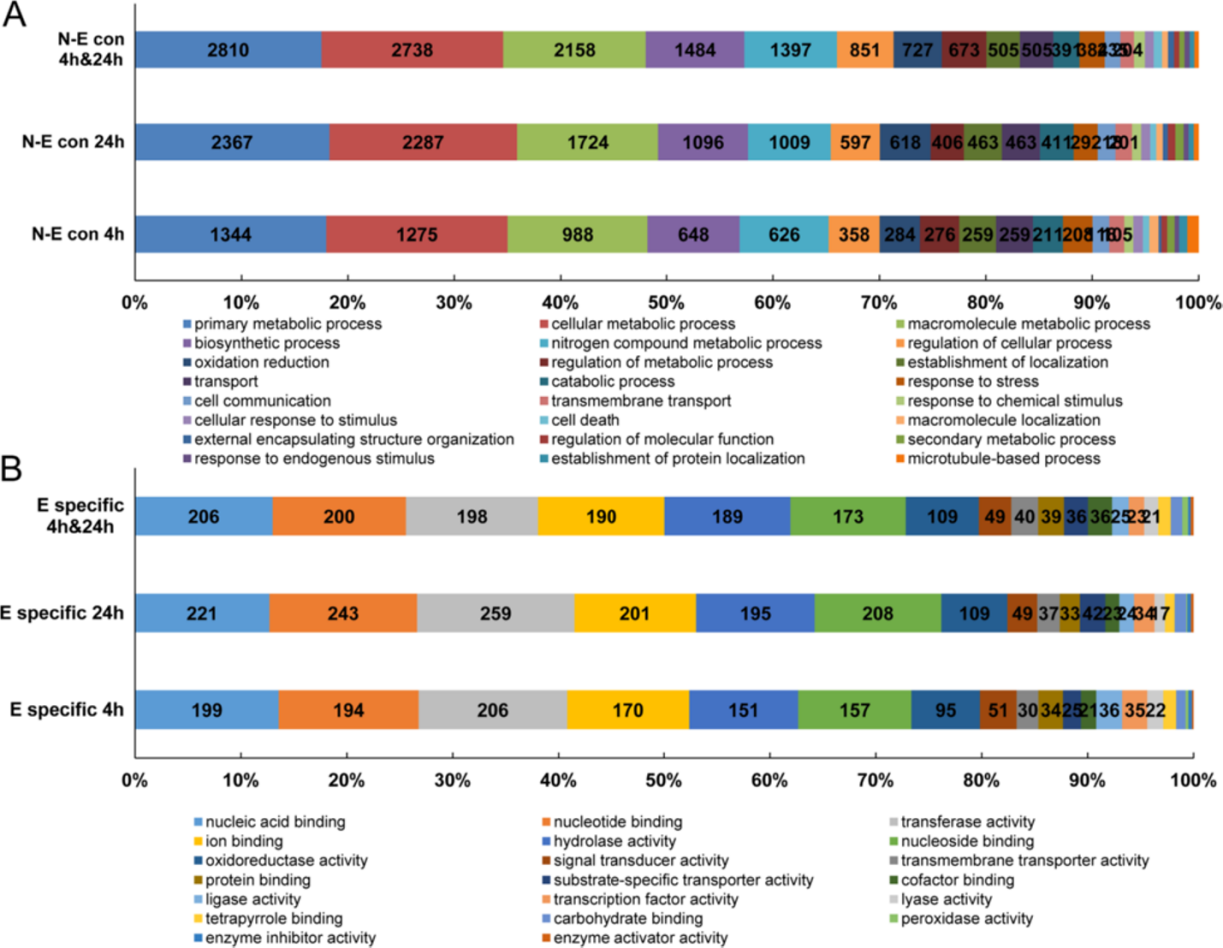
**Gene Ontology (GO) classification of six differentially expressed unigene (DEU) data sets in response to 200 mM NaCl stress for 4 and 24 h. (A)** Distributions of Nan Dan Ba Di Da Hua and Earlistaple 7 commonly DEU sets identified in four comparisons into GO biological process categories; **(B)** Distributions of Earlistaple 7-specfic responsive DEU sets identified in four comparisons into GO molecular function categories.

All differentially co-expressed unigenes were classified into three categories according to the significantly different expression patterns identified (Table 5). We also performed a STEM cluster analysis of all DEUs from the Earlistaple 7 genotype (Table 6), which revealed six distinct groups: early-stage up-regulated genes (Group I), late-stage up-regulated genes (Group II), continuously up-regulated genes (Group III), early-stage down-regulated genes (Group IV), late-stage down-regulated genes (Group V), and continuously down-regulated genes (Group VI). GO categories were assigned to DEUs in the six data sets (i.e., unigenes expressed in both genotypes at 4, 24, and both 4 and 24 h, and unigenes specifically expressed in Earlistaple 7 at 4 , 24, and both 4 and 24 h) (Figure 4). In the biological process category, GO classification of DEUs in the three data sets of expressed unigenes that were common to both genotypes at 4, 24, and both 4 and 24 h was similar, but the number of commonly expressed unigenes between the two genotypes at 4 h was less than the number at 24 and both 4 and 24 h (Figure 4A). In the molecular function category, the four most abundant subcategories in the three data sets of Earlistaple-7-specific unigenes were nucleic acid binding, nucleotide binding, transferase activity, and ion binding (Figure 4B).

**Table 5:** **Summary of the numbers of differentially co-expressed unigenes from the cluster 1 and cluster 2 by STEM analysis**

	Categories	ck, 4 h	ck, 24 h	ck, 4 h, 24 h	Total
N-E common		16,180	24,137	32,602	**72,919(78.2%** **)** ^**d**^
unigenes number^a^	Cluster 1	3,459(55)^c^	5,311(121)	5,051(315)	13,721(491)
	Cluster 2	4,102(60)	7,069(105)	7,922(163)	19,193(328)
E-specific		5,619	6,754	7,918	20,291(21.8%)
Unigenes number^b^	Cluster 1	997(15)	1,365(40)	1,095(30)	3,457(85)
	Cluster 2	1,331(27)	1,394(8)	1,541(9)	4,266(44)
All		21,799	30,891	40,420	93,210
Unigene numbers	Cluster 1	4,456(70)	6,6767(161)	6,146(345)	17,178(576)
	Cluster 2	5,433(87)	8,463(113)	9,463(172)	23,339(372)

Note: ^a^N-E common unigenes means the common differentially expressed unigenes between Nan Dan Ba Di Da Hua and Earlistaple 7.

^b^E specific unigenes means the specific DEUs expressed only in the Earlistaple 7.

^c^Numbers in parentheses indicate the number of TF unigenes.

^d^The percentage of differentially expressed unigenes in both genotypes before STEM analysis.

**Table 6:** **The classification and expression pattern of the identified differentially co-expressed unigenes**

Groups	I ck,4 h	II ck,24 h	III ck,4 h,24 h	IV ck,4 h	V ck,24 h	VI ck,4 h,24 h
Expression pattern						
Definition	Early induced up-regulated	Late induced up-regulated	Continuous induced up-regulated	Early induced down-regulated	Late induced down-regulated	Continuous induced down-regulated
N-E common unigenes^a^ no.	3,459(55)^**c**^	5,311(121)	5,051(315)	4,102(60)	7,069(105)	7,922(163)
E specific unigenes^b^ no.	997(15)	1,365(40)	1,095(30)	1,321(27)	1,394(8)	1,541(9)

^a^N-E common unigenes means the Nan Dan Ba Di Da Hua and Earlistaple 7 are common to differentially co-expressed unigenes.

^b^E special unigenes means the differentially co-expressed unigenes expressed only in the Earlistaple 7 genotype.

^c^Numbers in parentheses indicate the number of TF unigenes.

### Analysis of salt stress-responsive TFs and other salt tolerance-related functional genes

Although expression patterns of many unigenes were similar between Earlistaple 7 and Nan Dan Ba Di Da Hua, 78.2% of salt-responsive unigenes identified in Earlistaple 7 were more or less differentially expressed (Nominal false discovery rate (FDR) < 0.001) in Nan Dan Ba Di Da Hua at one or more time points (Table 5). TFs are important regulators of gene expression. The related the expression abundance and annotation information of TF unigenes in the six data sets by STEM analysis were obtained (see Additional file 2C-H).

An overlap was only observed in TF unigenes responsive to NaCl stress between Earlistaple 7 and Nan Dan Ba Di Da Hua at the 4-h time point, with 55 TFs transcriptionally up-regulated and 60 down-regulated (Table 6). These differentially expressed TFs belonging to AP2-EREBP family were followed in abundance by WRKY, NAC, MYB, and C2H2 under 4- and 24-h NaCl treatment (see Additional file 2C–E). It should be noted that all of these DEUs were expressed in the salt-tolerant genotype Earlistaple 7, but were repressed, weakly induced, or not induced at all in salt-sensitive Nan Dan Ba Di Da Hua (see Additional file 2I and Figure 3C). Some of these weakly/non-induced genes, such as *GhNAC4*, *GhNAC*5, *GhWRKY2*, *GhERF5*, and *GhDREB1L*, play very important roles in salt tolerance of cotton (Table 7).

**Table 7:** **The specific transcription factor unigenes response to salt stress in Earlistaple 7**

Unigene ID	Accession	Annotation	E-value	Earlistaple 7 Log2 ratio	
				4 h	24 h
comp1995_c0_seq1	Q9FWX2	NAC domain-containing protein 7	7E-51	-2.00	-1.52
comp105823_c0_seq1	Q39261	Zinc finger protein 2	9E-22	-1.63	-1.39
comp100893_c1_seq1	Q9ZWM9	AP2/ERF and B3 domain transcription factor RAV	8E-78	-1.01	-1.65
comp109426_c0_seq4	Q9FLX8	Probable WRKY transcription factor 27	9E-58	-0.56	-2.14
comp1104709_c0_seq1	Q39265	Zinc finger protein 6	2E-25	0.00	-1.53
comp534425_c0_seq1	B9SUH9	CCAAT-binding transcription factor. Putative	2E-11	0.00	-1.58
comp94181_c0_seq1	Q688R3	Zinc finger CCCH domain-containing protein 33	1E-16	1.05	2.14
comp105064_c1_seq2	Q9XEE6	Zinc finger CCCH domain-containing protein 29	6E-79	1.07	2.10
comp77518_c0_seq1	E2FGB5	Homeodomain-leucine zipper protein HD2 GhHB2	1E-53	1.92	3.29
comp103962_c0_seq1	Q6VY01	Putative dehydration responsive GhDREB1A	3E-53	2.26	3.62
comp87977_c0_seq1	Q9LR65	Probable protein phosphatase 2C 1	1E-69	2.47	2.35
comp64928_c0_seq1	B9GYM6	Ethylene-responsive transcription factor ERF105	6E-07		2.18
comp97922_c0_seq1	Q9XEE6	Zinc finger CCCH domain-containing protein 29	4E-91		2.08
comp96688_c0_seq1	Q8LCG7	Nuclear transcription factor Y subunit C-2 NFYC2	3E-26		1.98
comp72703_c0_seq1	Q9SV15	Probable WRKY transcription factor 11	7E-27		1.98
comp108958_c0_seq1	B9SRT4	WRKY transcription factor. Putative	1E-65		1.89
comp74963_c0_seq2	Q9XJ60	MADS-box transcription factor 50	1E-30		1.85
comp106969_c0_seq1	Q6X7J9	WUSCHEL-related homeobox 4	5E-60		-1.2
comp67684_c0_seq1	Q9FJV5	Probable transcription factor KAN4	3E-09		-1.48
comp81996_c0_seq1	A4L9W4	Auxin response factor 3 GhARF3	9E-16		-2.14
comp114981_c0_seq2	Q9FHH8	Zinc finger protein CONSTANS-LIKE 5	3E-23		-2.15
comp426377_c0_seq1	A9PL22	Homeobox protein GhHB1	1E-58		-2.88
comp79059_c0_seq1	Q9SK55	NAC domain-containing protein 42	3E-77	-4.11	
comp60998_c0_seq1	Q681X4	Zinc finger protein ZAT5	6E-16	-2.55	
comp71059_c0_seq1	O80933	Scarecrow-like protein 9	5E-41	-2.45	
comp107981_c0_seq1	B9SMN9	Transcription factor. Putative	7E-33	-2.41	
comp98436_c0_seq1	D3XFF8	TT2 like MYB transcription factor	2E-77	-2.26	
comp398_c0_seq1	O04291	Homeobox-leucine zipper protein ATHB-14	3E-25	-2.15	
comp108793_c0_seq1	Q8LAP8	Dof zinc finger protein DOF4.6	2E-38	-2.1	
comp2403_c0_seq1	Q9SB61	ZF-HD homeobox protein At4g24660	2E-41	-1.84	
comp55428_c0_seq1	G7JVA8	Zinc finger protein-like Ser/Thr protein kinase-like protein	5E-75	-1.44	
comp69223_c0_seq1	Q9LHJ9	Probable protein phosphatase 2C 38	2E-142	1.63	
comp67435_c0_seq1	B9RA11	Transcription factor. Putative	2E-26	1.71	
comp60938_c0_seq1	E6Y3E2	WRKY transcription factor PmWRKY110 (Fragment)	1E-11	1.96	
comp798487_c0_seq1	Q4ZJA9	BZIP-like protein *Gossypium hirsutum*	2E-19	2.5	

Under NaCl stress, most of the salt-responsive TF unigenes exhibited genotype specificity. We identified 129 TF unigenes whose expressions were specific to Earlistaple 7, of which 85 were up-regulated and 44 were suppressed under salt stress (see Additional file 2 F-H). These 129 TF unigenes, which corresponded to 1.6% of the 8,127 salt-responsive genes of Earlistaple 7, were only expressed during either early or late stages of salt stress. We note that some TF unigene families expressed under both 4- and 24-h salt stress were identical to the above-mentioned TF families commonly expressed in both genotypes, but the expressed TF was different. The expression trend of the WRKY TF unigene (homologous to *PtWRKY25*) was different at the two time points—decreasing at the beginning and then increasing, while that of others, such as AP2-EREBP, MYB, HB, NAC, WRKY, and bHLH family members, experienced a continual rise.

Many other salt tolerance-related unigenes encoding transporters, Ca^2+^ binding protein, heat-shock proteins (HSPs), detoxificants, and dehydration-response proteins were differentially expressed under 4- and 24-h salt stress (see Additional file 4). Several differentially expressed genes encoded transporters contributing to the re-establishment of ionic and osmotic homeostasis under salt stress, such as ABC transporters (i.e., ABC transporter B, C, and G family members), sodium/hydrogen exchanger (NHX1), ATPases (Ca^2+^-ATPase, H^+^-ATPase, and V-type H^+^-transporting ATPase), potassium transporter, and nitrate transporter. Genes encoding some Ca^2+^ sensors, namely CML and CPK, and one CBL-interacting protein kinase (CIPK) gene were identified in the different libraries. Two cotton CBL genes (*GhCBL1* and *GhCBL8*) were highly expressed under 4- and/or 24-h salt stress in Earlistaple 7. Unigenes encoding CDPK and CIPK members were up-regulated under 4- and/or 24-h salt stress in both genotypes. Genes from several HSP families (e.g., HSP20, HSP70, Hsp90, and DanJ) as well as genes related to reactive oxygen species (ROS) scavenging and detoxification (CAT, POD, GPX, ALDH, and RBOH) and dehydration response (LEAs and RING/U-box domain-containing protein/XERICO) were highly up-regulated at different salt-treatment time points either in Earlistaple 7 or Nan Dan Ba Di Da Hua (see Additional file 4). Finally, genes involved in salt-stress tolerance pathways, such as CDPK, starch and sucrose metabolism, carbohydrate metabolism, and MAPK signal pathways, were found to be induced at different time points. These identified unigenes are potentially salt-induced genes and may serve as a supplementary resource for salt-tolerance gene mining.

**Figure 5:**
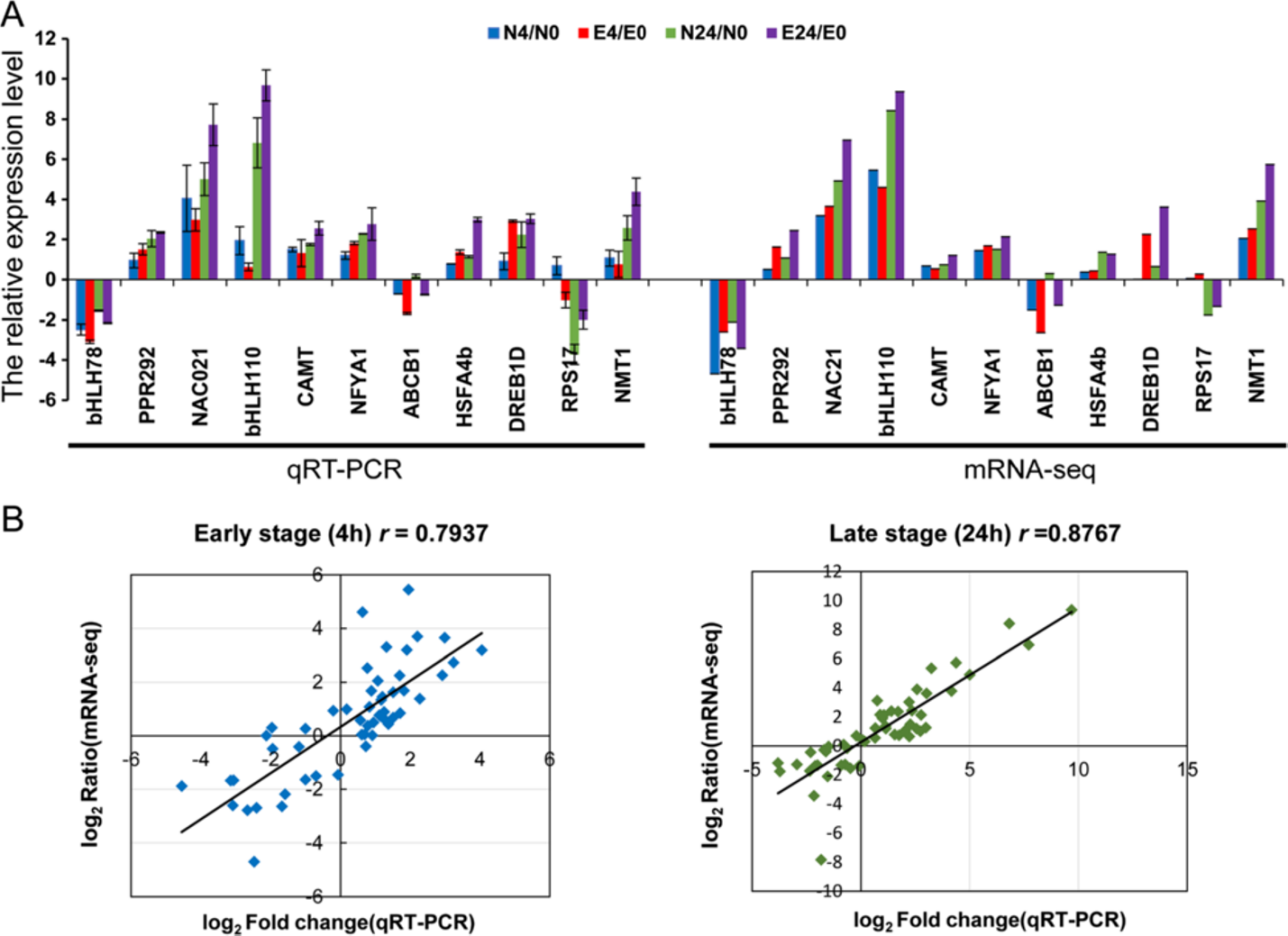
**Quantitative RT-PCR validation of randomly selected unigenes. (A)** qRT-PCR validation of the expression patterns of 11 representative unigenes from different comparisons. The *y-axis* represents qRT-PCR relative expression levels from three independent biological replicates (left *x-axis*) and the log_2_ fold-change of the unigene (right *x-axis*). **(B)** Correlation analysis of 26 highly differentially expressed unigenes in leaves under salt stress for 4 and 24 h based on qRT-PCR and mRNA-seq data; Pearson correlation coefficients (r) are 0.7937 and 0.8767, respectively (*P* < 0.001).

To further validate the results of mRNA sequencing, 26 transcripts were randomly selected along with their specific primers for quantitative real-time PCR (qRT-PCR) analysis (see Additional file 5A). Eleven of the 26 tested transcripts had expression patterns identical to those observed in the mRNA-seq experiments (Figure 5A). Expression levels measured by qRT-PCR were strongly correlated with those of DEUs identified by mRNA-seq (*r =* 0.793 and 0.877 under 4- and 24-h salt stress, respectively), demonstrating the reliability of the mRNA-seq results (Figure 5B and Additional file 5D).

### Deep sequencing of small RNAs and identification of conserved miRNAs under salt stress

To characterize differences between Nan Dan Ba Di Da Hua and Earlistaple 7 at the miRNA regulatory level and to identify conserved miRNAs in cotton under salt stress, we constructed and analyzed six small RNA libraries of the two genotypes. A total of 48,585,151 sequence reads ranging in length from 17 to 35 nt were generated. After removing adapter sequences, low-quality tags, and reads shorter than 18 nt, we obtained 22,681,645 clean reads, which corresponded to more than 2 million unique sequences for each sample (see Additional file 6A). As shown in Additional file 6B, most small RNAs were 20–24 nt in size; the 24-nt class was the most heavily represented (50.56%) in the Nan Dan Ba Di Da Hua small RNA control library, followed by 23-nt (7.37%), 21-nt (10.27%), and 22-nt classes (7.14%).

The six libraries of clean reads were compared against tRNAdb, SILVA rRNA, and NONCODE v3.0 databases, allowing for mismatches. Detailed results of this comparison are shown in Table 8. After annotation and removal of non-coding small RNAs, 320 conserved miRNAs were identified and grouped into 37 miRNA families, with those belonging to unknown families designated by “NA” Comparison of the six libraries against the miRbase19.0 database, which contains 39 known ghr (*G. hirsutum*) miRNAs, identified 21 conserved miRNAs belonging to 17 families, including three families that are cotton-specific: miR2948 (ghr-miR2948-5p), miR2949 (ghr-miR2949a-5p, ghr-miR2949a-3p, and ghr-miR2949b), and miR3476 (ghr-miR3476-5p) (see Additional file 7).

**Table 8:** **Distribution of sequence reads among different RNA categories in six libraries of cotton**

	Sample	All	rRNA	snoRNA	tRNA	snRNA	miRNA	Unanotation
Unique sRNA number	N0	440,466	32574.5	153.5	283	235	626(0.14%)	406,594
	N4	431,000	36,940	166	297	249	650(0.15%)	392,698
	N24	339,460	36,430	101	272	257	508(0.15%)	301,892
	E0	237,949	25,002	94	176	107	479(0.20%)	212,091
	E4	365,569	34,035	133	198	253	449(0.12%)	330,501
	E24	807,866	33,293	64	134	90	376(0.05%)	773,909
Total sRNA number	N0	4,226,267	1,073,632	1705	3596	1165	320,413(7.58%)	2,825,756
	N4	4,208,092	1,207,710	1812	3603	1065	335,427(7.97%)	2,658,475
	N24	4,821,011	1,395,936	1782	3923	1656	379,404(7.87%)	3,038,310
	E0	2,378,551	626,216	813.5	2138	368	327,868(13.78%)	1,421,148
	E4	2,999,177	814,357	1250	2197	918	335,427(11.18%)	1,845,028
	E24	4,048,547	461,580	236	1702	234	73,340(1.81%)	3,511,455

The number of identified miRNAs and the abundance of miRNA families are listed in Additional file 6C–D and Additional file 7. The reads for each miRNA family were normalized as “reads per million mapped reads” (RPM). Among miRNA families, miR166 exhibited the highest abundance (3,909,410 RPM). Other highly expressed families, including miR396, miR482, miR159, miR1697, miR394, and miR156, were represented by approximately 100,000 RPM, whereas miR319, miR2118, miR858, miR828, miR829 and miR1310 families had less than 100 RPM. Differences among miRNA families were also revealed by the number of members they contained (see Additional file 6D and Additional file 7). miR166 was the largest family, with 29 members, while miR159, miR396, miR171, and miR156 possessed 28, 26, 23, and 20 members, respectively. Different family members also displayed drastically different expression levels (see Additional file 7). In the miR156 family, for instance, RPM of ghr-miR156a was 308 in the control Nan Dan Ba Di Da Hua library, while the RPM of cca-miR156b was only 2.6.

**Figure 6:**
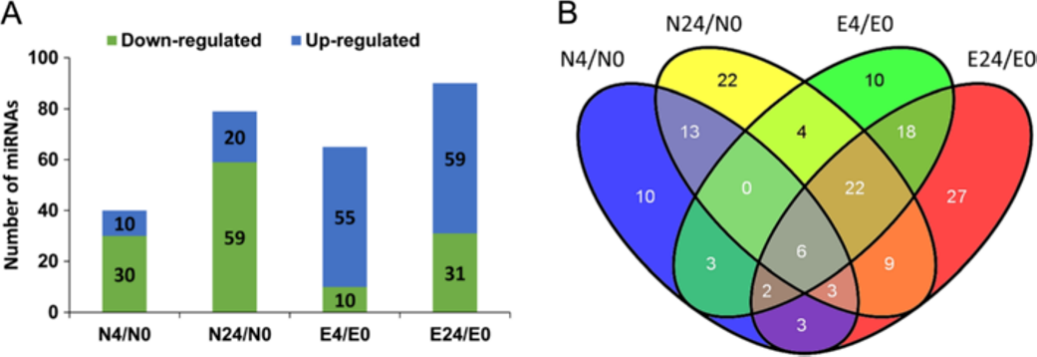
**Abundances of differentially expressed conserved miRNAs identified in the two genotypes under 4- and 24-h salt stress. (A)** Number of differentially expressed miRNAs (*P* ≤ 0.05 and |log_2_ Ratio| ≥1) compared with their respective control samples; **(B)** Venn diagram illustrating the number of congruously and oppositely regulated miRNAs between Nan Dan Ba Di Da Hua and Earlistaple 7 in response to salt stress at various time points.

We identified miRNAs that were differentially expressed (*P* < 0.05 and |log_2_ Ratio| ≥ 1) between treatment and control (RPM of 4- and 24-h NaCl treatment compared with RPM of control) from 37 families (see Additional file 8). As shown in Figure 6A, the differentially expressed miRNAs were transcriptionally regulated at different time points in the two genotypes under salt stress. We detected both congruously and oppositely regulated miRNAs as well as those that were genotype-specific (Figure 6B and Additional file 8). Further analysis revealed that most differentially expressed miRNAs displayed different expression patterns between the two genotypes or the two time points (see Additional file 9A, B). These results demonstrate that miRNA expression changes in cotton under early-(4 h) and late-stage (24 h) salt stress are similar, and that differential regulation of miRNAs may be responsible for the distinct salt sensitivities of the two cotton genotypes.

### Combined analysis of conserved miRNAs and their target genes under salt stress

**Figure 7:**
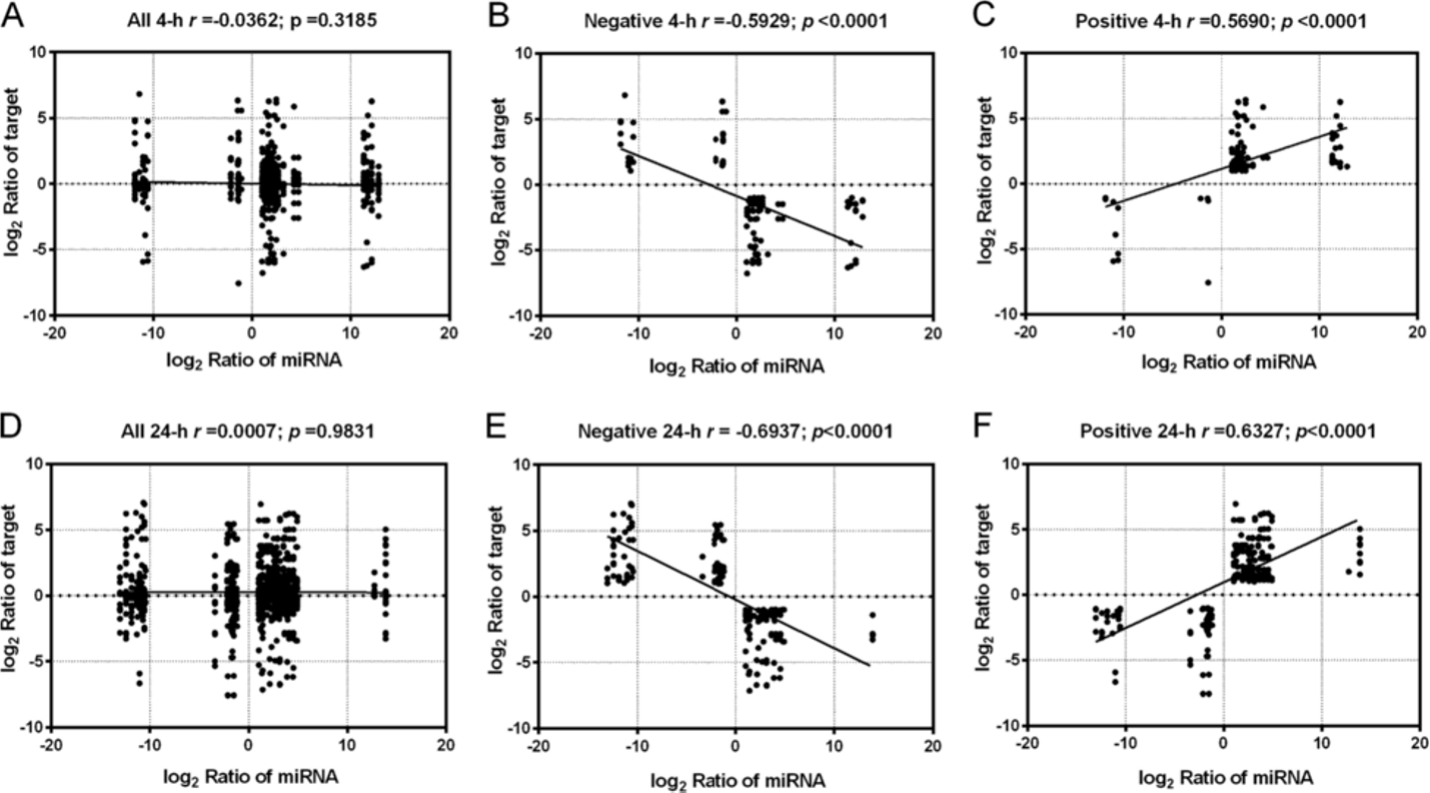
**A combined view of correlated expression between a miRNA and its target in cotton under early- and late-stage salt stress.** Pearson correlation coefficients (PCCs) between the expression of miRNAs and miRNA targets were calculated. Black circles represent miRNA and target pairs. PCCs are represented by the linear slope and are listed numerically, with **A**, **B** and **C** indicating all, negative, and positive correlations for 4-h, respectively. The same means with **D**, **E** and **F** for 24-h.

To discover salt tolerance-related miRNAs in cotton, we used the analytical approach shown in Additional file 9C. We uncovered 108 differentially expressed miRNAs and compared their expressions in the two genotypes (see Additional file 8E). At least one comparison (E4/E0 or E24/E0) showed significant changes at the *P* < 0.05 level. By incorporating the transcriptome sequencing results into the comparisons E4/E0 and E24/E0 for all 108 differentially expressed miRNAs under salt stress, we detected 761 and 1,016 miRNA-target pairs, respectively. Generally, microRNAs negatively regulate the accumulation of their target mRNAs therefore when they are expressed in common tissue, their expression profiles should show negative correlation [34]. But [52] found an equal number of positively and negatively correlated miRNA/target pairs indicating that positive correlation is more frequent than previously thought [52]. In our research, we attempted to reveal the molecular regulation mechanism of salt tolerance in upland cotton, microRNA might played an important role during the response process. Therefore, the first step was to found out the correlationship between miRNA expression and target mRNA expression, we calculated Pearson correlation coefficients (PCCs) between miRNAs and their targets. The PCCs between all 761 and 1,016 miRNAs and their target pairs were -0.0362 and 0.0007 at 4-h and 24-h in Earlistaple 7, respectively, indicating no correlations (Figure 7A, D). Then we found these miRNA were grouped into three categories: positively correlated, negatively correlated, and not correlated according to their target gene expression. It is obviously the no significant correlation between total miRNAs and their targets maybe result from the opposite effects of the positively and negatively correlation of the miRNA/target pairs. Therefore, we should separate the positively correlated and negatively correlated miRNA/gene pairs for further analysis based on the previous studies [52], The PCC and linear regression analysis of the positively and negatively correlated miRNA/gene pairs were shown in Figure 7B, C, E, F. These results suggested that negative correlation is similar to the positive correlation and complex regulatory networks exist between miRNAs and their targets in cotton under salt stress. Finally, after excluding unannotated target unigenes from E4/E0 and E24/E0 comparisons, we respectively obtained 65 and 101 negative miRNA-target gene relationships to further analysis (see Additional files 9D and 10A,C). When GO categories were assigned to these targets, six molecular function categories predominated; among these, “hydrolase activity”, “nucleoside binding”, and “nucleotide binding” were the most highly represented. Fourteen biological processes were identified, the most frequent being “cellular metabolic process” and “primary metabolic process”. With respect to cellular components, seven classes were uncovered, with “intracellular part” and “cell part” the most abundant (see Additional file 9E). These classifications suggest that miRNA targets associated with salt tolerance are primarily related to binding, catalytic activity, cellular metabolic, and other metabolic processes.

**Figure 8:**
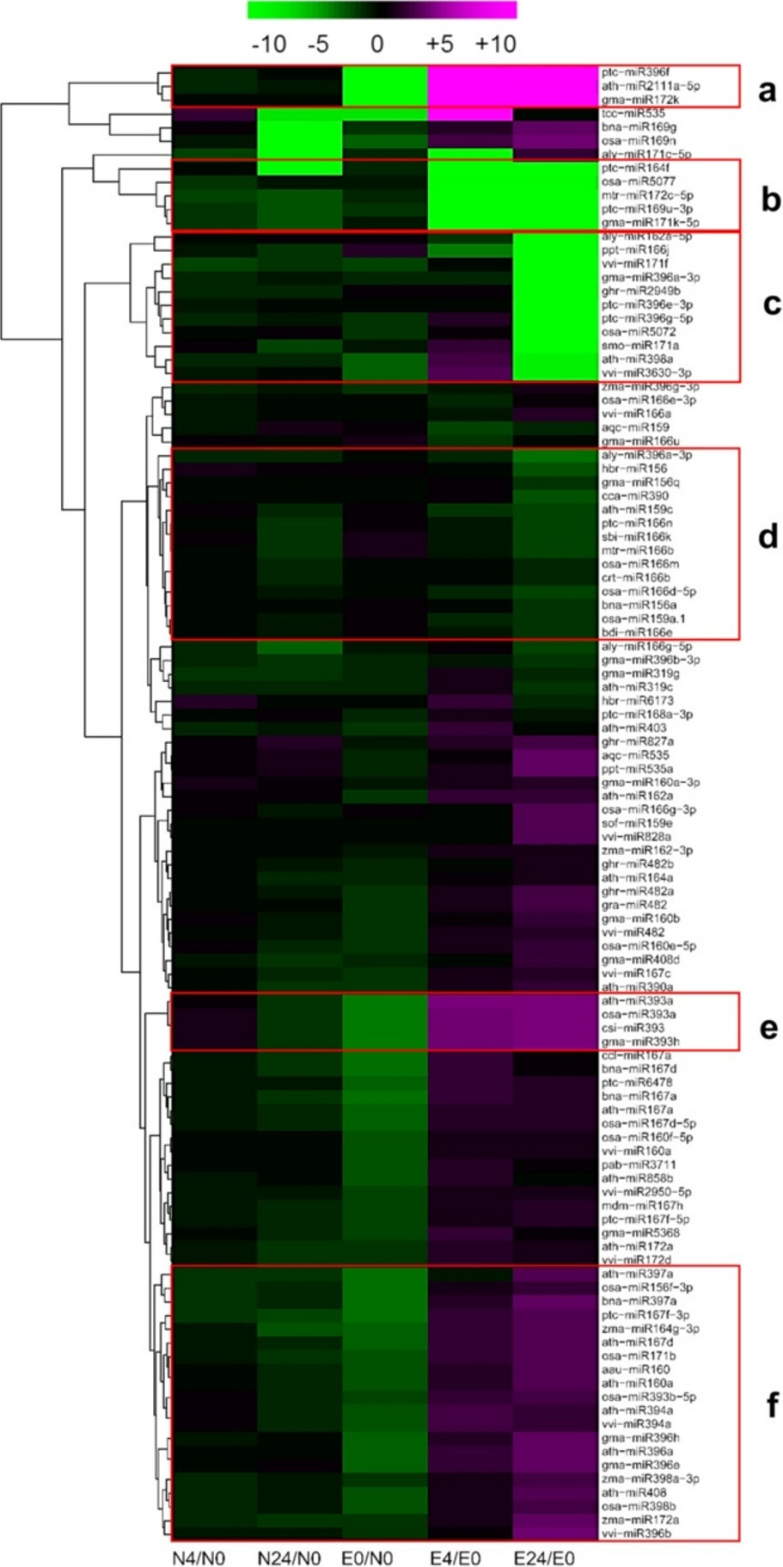
**Complete linkage hierarchical cluster analysis of 108 differentially expressed miRNAs in salt-tolerant Earlistaple 7.** Log_2_ Ratio was indicated on a color scale from magenta (high) to green (low). miRNA names are on the right side of the figure, and the five comparisons of both genotypes under 4- and 24-h of salt stress were at the bottom; The separated 57 representative conserved miRNAs were divided six clusters as following **a**, **b**, **c**, **d**, **e**, **f** to further analysis. The miRNA name, log2 Ratio for each cluster are presented in Additional 11B.

To elucidate the potential regulatory roles of miRNAs in cotton under salt stress, we analyzed differential expression profiles of 108 miRNAs in Earlistaple 7 and Nan Dan Ba Di Da Hua (see Additional file 11). According to the results of hierarchical cluster analysis, 57 of these representative conserved miRNAs exhibited significant differential expression during salt stress and were selected six clusters (a, b, c, d, e, f) were selected from a whole sub-branch by hierarchical cluster analysis which indicated they presented a very similar expression pattern (Figure 8). To better understand the functions of the 57 representative conserved miRNAs and seven specifically expressed conserved miRNAs, we predicted their putative targets (see Additional file 11C,D,E).

**Figure 9:**
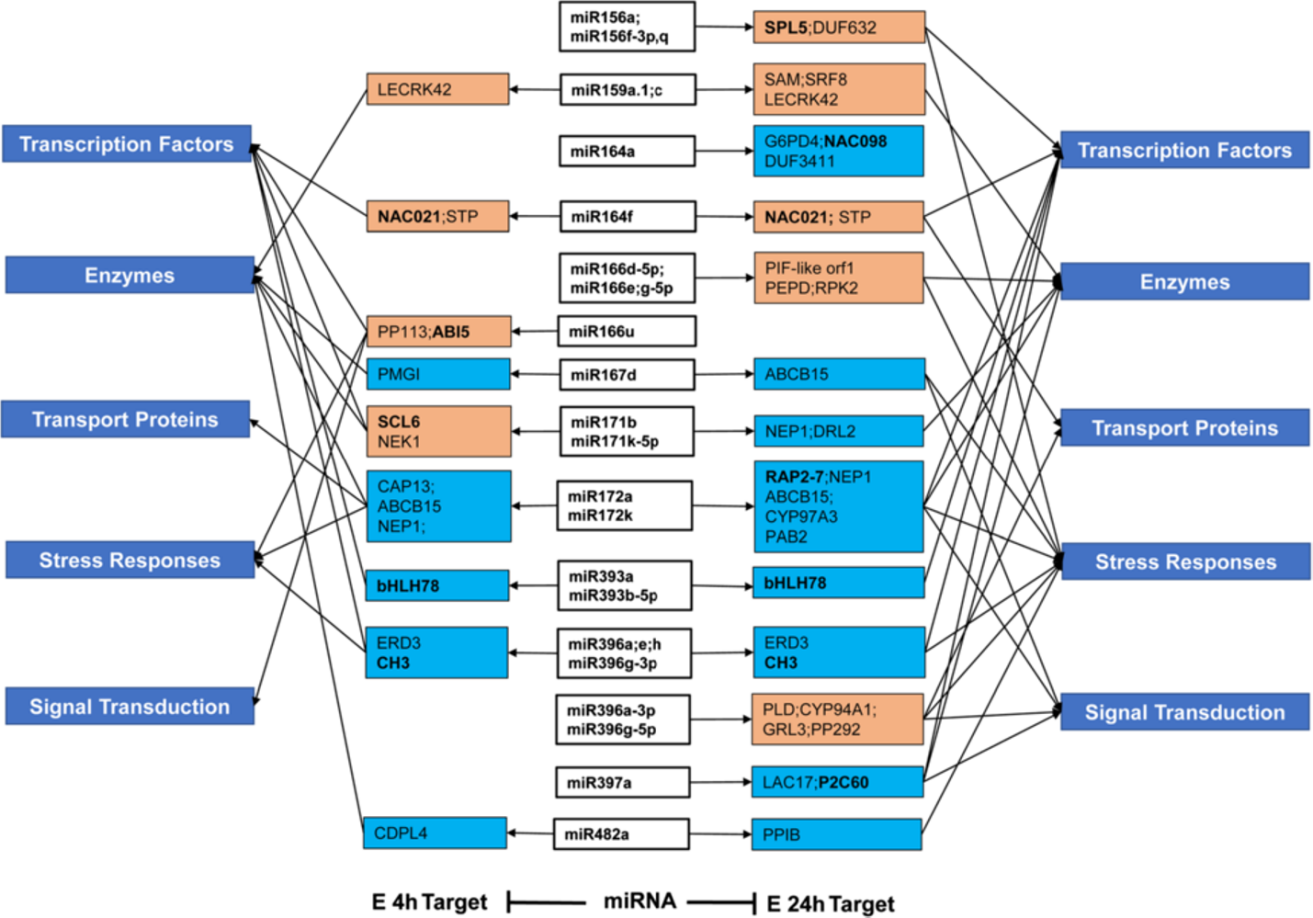
**A proposed regulatory network of salt-responsive conserved miRNAs in cotton leaves.** The data contributing to the network were collected from Earlistaple 7 leaves treated with 200 mM NaCl for 4 and 24 h, and show the potential roles of salt-responsive miRNAs in early stages of salt stress. The color of the miRNA target box indicates the type of expression pattern and is consistent with the data shown in Additional file 11E; orange indicates up-regulated miRNA targets, lake blue indicates miRNA targets with gradually reduced expression. TF genes in target boxes are indicated in bold. Arrows between miRNAs and target genes indicate negative regulation. Target gene types are indicated by sky blue boxes.

We integrated information about the 64 conserved miRNAs into the E4/E0 and E24/E0 miRNA-target pair comparisons, which yielded 75 miRNA-target pairs for 40 conserved miRNAs from the six groups (Additional file 11C). The miRNA-target regulatory networks inferred at different time points (4 and 24 h) are shown in Figure 9. Several conserved miRNAs (including miR156, 164, 166, 171, 172, 393, 396, and 397) were predicted to target potential TF genes such as those encoding squamosa promoter binding protein (*SPL5*), an NAC-domain transcription factor (*NAC021*), a bZIP protein (*ABI5*), an MYB transcription factor, Scarecrow-like protein (*SCL6*), AP2 domain-containing protein, Basic helix-loop-helix (*bHLH78*), an WRC domain (*C3H*), ethylene-responsive transcription factor (*RAP2-7*), and protein phosphatase 2C (*P2C60*). A total of 19 and 34 target genes related to 33 conserved miRNAs were induced in the salt-tolerant line Earlistaple 7 after 4 and 24 h of salt stress, respectively (Figure 9 and Additional file 11).

**Figure 10:**
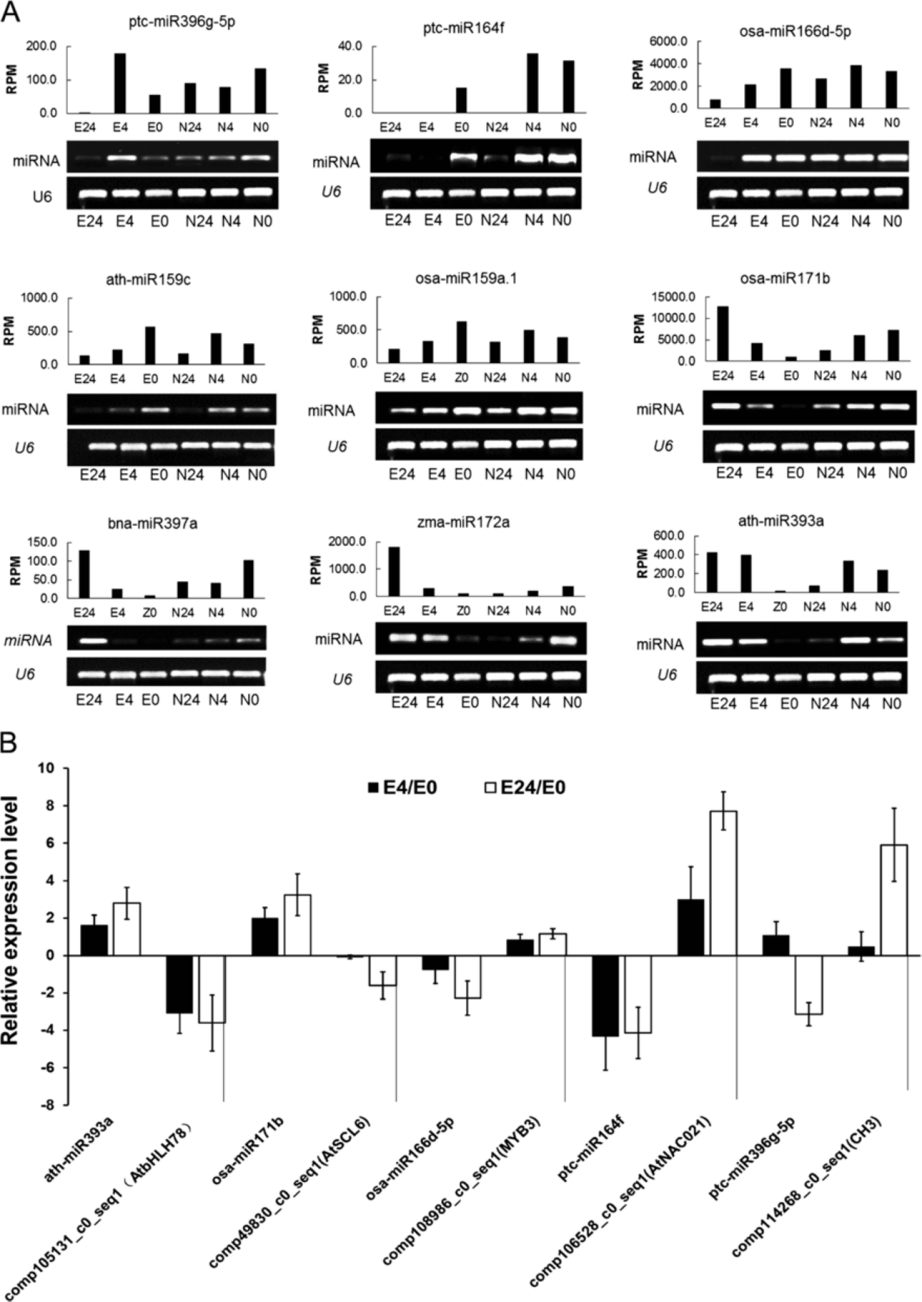
**Expression profiles of nine cotton salt-responsive miRNAs and their potential corresponding targets detected by sequencing, stem-loop RT-PCR, and qRT-PCR assays. (A)** Stem-loop RT-PCR verification of the expression patterns of nine conserved miRNAs in Nan Dan Ba Di Da Hua and Earlistaple 7 leaves after 4- and 24-h salt stress. The upper bar chart shows the relative expression of conserved miRNAs obtained by sequencing; the lower electropherogram indicates the relative expression levels of miRNA based on RT-PCR. **(B)** A combined view of inverse expression between a miRNA and its potential target in Earlistaple 7 under salt stress for 4 and 24 h according to qRT-PCR and stem-loop RT-PCR. miRNA expression (left side) was validated by sequencing of small RNAs isolated from control (ck) and salt-treated (4 and 24 h) samples. The miRNA targets (right side) were validated by transcriptome sequencing, and their expressions were checked by qRT-PCR. Up- or down-regulation in expression was normalized to U6 snRNA or *Actin* levels.

The expression patterns of nine conserved miRNAs were validated by stem-loop RT-PCR (Figure 10A and Additional file 5B). As expected, the stem-loop RT-PCR data showed a high degree of consistency with the expression profiles obtained by small RNA sequencing. These data also revealed that two miRNA targets were negatively regulated by their miRNAs. As shown in Figure 10B, at-miR393a expression increased between 4 and 24 h of salt treatment, whereas comp105131_c0_seq1 *(*homologous to *AtbHLH78*) expression decreased, with its transcript levels negatively correlated with the accumulation of at-miR393a. Relative expression of ptc-miR164f was reduced to low levels at 4 and 24 h of salt treatment, while the expression level of its target gene (homologous to *AtNAC021*) remained unchanged, compared with the controls, during this period.

## Discussion

### Prerequisites for identification of candidate salt tolerance-related genes and miRNAs in cotton

Two main factors are responsible for salt stress-induced inhibition of plant growth: osmotic stress and ionic stress [50]. Different mechanisms control development at different time points in plants exposed to salinity. In this study, we monitored the physiological responses of two cotton genotypes with differing salt tolerances at two distinct phases. We found that these phases were characterized by increases or decreases of clusters of differentially expressed genes and miRNAs.

The positive relationship between water content and Na^+^ concentration causes ions to accumulate in vacuoles, which lowers water potential, increases the driving force for water uptake, and increases turgor [53]. Maintenance of K^+^ and Na^+^ homeostasis is crucial under salt stress [54]. Our study results suggest that after being taken up by the roots, Na + is transported to shoots through the stems and the roots of salt-tolerant genotype Earlistaple 7 were able to retain more Na^+^ than Nan Dan Ba Di Da Hua (Figure 1D, E). To avoid excessive ion accumulation in roots owing to continued Na^+^ uptake, compensatory decreases in other cations occurred in the roots at 24 h. Shoot leaves accumulated Na^+^ in addition to the cations already present, with the tolerant genotype thereby experiencing the strongest ionic stress beginning at 24 h (Figure 1F).

By examining these dynamics, we gained insights into physiological responses of the NaCl-acclimation process, which consisted of an initial dehydration phase in leaves (0–4 h) and subsequent NaCl accumulation (4–24 h) followed by restoration of osmotic homeostasis at a new ionic stress level (24 h) and either final adjustment to a steady ion balance or ion-induced damage (24–72 h and beyond). The timing of these adaptive responses differed between roots and leaves. In previous studies, either roots or leaves were used to identify differences between early and late transcriptional responses to salt treatment in cotton [31–34]. Our study of two genotypes with contrasting salt tolerances revealed that Na^+^ accumulated mainly in shoots, and that significant physiological and morphological differences occurred at different time points under salt stress: 4 h (owing to dehydration stress) and 24 h (owing to ionic stress). These two important time points thus corresponded to the two typical phases of plant growth response to salt stress.

In some studies, genes differentially expressed in response to salt treatment have been identified using a single plant genotype [47–49]. Two or more genotypes with contrasting salt tolerance have only been used in a few reported investigations [46, 55–57]. To identify genes responsible for salt tolerance, analysis of genotypes with similar genetic backgrounds and contrasting salt tolerances is preferable. Identification of common genes that are differentially expressed under stress conditions between tolerant and sensitive genotypes with different genetic backgrounds has been suggested as an alternative approach [58]. As pointed out in [58], it is impossible to isolate stress tolerance-related genes from stress-responsive ones unless transcriptional-level differences are compared between tolerant and sensitive genotypes under stress conditions [58]. Genes related to salt tolerance can thus be identified in genotypes with different genetic background and contrasting tolerances by comparative analysis of differentially expressed genes and miRNAs at different salt-treatment time points. We therefore used a pair of salt-tolerant and salt-sensitive genotypes in this study to identify differentially expressed genes and miRNAs.

### Abundance of differentially expressed TF unigenes in response to salt stress in tolerant and sensitive genotypes

To govern necessary transcriptional regulation in response to developmental and environmental conditions, plant genomes encode a large number of TFs. In the present study, we identified 3,202 TF unigenes (see Additional file 2A) by global TF classification of DEUs. Many TFs in the AP2/EREBP family, followed to a lesser extent by WRKY, MYB, NAC, DBP, bHLH, C2H2, C3H, C2C2, and CCAAT family members, were up-regulated during salt stress (Figure 2 and Additional file 2B). Some members of these families have been reported to participate in abiotic stress response, suggesting their roles in plant adaptation to stress [18, 59–62]. Seven unigene clusters were significantly assigned as comprising co-expressed genes according to STEM (Figure 3A). The mechanism responsible for these gene co-expression clusters is unclear, although the co-expression of clustered genes may be partially regulated by the interaction of common elements and TFs [63]. A previous promoter analysis has suggested that some of these co-expressed genes may be regulated by common TFs [64]. In light of the above views, we separated out the differentially co-expressed unigenes from all differentially expressed genes encoding TFs to identify the types of TFs regulating response to 4- and 24-h salt stress (Table 5 and Additional file 2C–H).

### Salt-responsive TF unigenes in the two cotton genotypes

Dramatic changes were observed in TF unigene abundance and types (common and specific) at 4- and 24-h salt-stress time points in the two contrasting genotypes. Among these changes, 478 TF unigenes (groups III and IV in Table 6) exhibited similar patterns of altered expression between the two genotypes. This dramatic variation is consistent with the plant switching from normal growth and development to stress-specific responses. Our observation is the first reported up-regulation of *GhbZIP, GhDREB1C, GhMYB, GhMYB2,* and *GhWAKY* under 4- and 24-h salt-stress treatment. *AtRAP2-12* and *AtRAP2-3*, homologs of cotton genes *GhERT* and *GbERF2*, are reported to increase rapidly in Arabidopsis under salt stress [65, 66]. Our results illuminate the roles of these genes in response to salt stress (in addition to abiotic stresses). In an earlier study, bHLH family member *GhbHLH1* was transitorily induced in leaves by abscisic acid (ABA) and polyethylene glycol treatments [67]. In our study, *GhbHLH1* was significantly up-regulated (see Additional file 2E), indicating its additional involvement in cotton salt-stress response. Seven TF genes (three *GhNAC*, two *GhHB* and two *GhMYB* genes) were identified at 4- and 24-h salt-stress time points, suggesting that their protein products may play an important role in salt-stress response in addition to the involvement in ABA signaling uncovered in previous research [22, 68, 69]. Transcripts encoding C2H2-type zinc finger proteins (ZAT10) that have been implicated in salt stress tolerance were up-regulated under 4- and 24-h salt stress. Overexpression of C2H2-type zinc finger proteins (*STZ/ZAT10* homologs) has been shown to induce the expression of several stress genes related to salt tolerance, dehydration, and cold stress [70, 71]. *GhMADS7* exhibited higher expression levels in the two cotton genotypes upon 4 and 24 h of salt treatment. Although GhMADS7 is a well-known participant in cotton fiber elongation, salt-stress response is a novel function for this TF; alternative splicing may have altered its cellular role [72].

Although 115 “osmotic stress”-specific TF unigenes were observed after 4 h of stress treatment, most of the 226 “salt stress”-specific TF unigenes appeared to be ion-specific at 24 h (groups I, II, IV, and IV in Table 5). These two salt-stress treatment phases clearly triggered very different responses in the two contrasting genotypes. Another finding of our study was that 37 unigenes belonging to the AP2/EREBP family of TFs were also related to salt tolerance. DREB/CBF subfamily genes, such as *DREB2A* [73, 74] and *DREB2B* [75], are reportedly involved in osmotic (4 h) and ionic (24 h) stress. Interestingly, we also found that some type-B ARRs (e.g., *AtPRR7*) were induced or repressed at 4- and 24-h time points (Additional file 2C, D). These general salt-responsive genes identified at 4 h (osmotic stress) are thus similar to those identified at 24 h (ionic stress). Because those unigenes were expressed in both tolerant and sensitive genotypes, they may not be directly responsible for salt tolerance.

### Salt-responsive TF unigenes in the salt-tolerant cotton genotype

We detected 129 unigenes—48 at 4 h, 42 at 24 h, and 39 at both time points (Table 5 and Additional file 2 F, G, H)—that were differentially expressed under NaCl treatment in the salt-tolerant genotype but not in the salt-sensitive one. In addition, unigene expression in the tolerant line Earlistaple 7 differed dramatically between 24- vs. 4-h salt stress (Table 5). One possible explanation for this temporal variation is that the expression of upstream response genes (including TFs) was gradually reduced during osmotic stress recovery, with the upstream cascade genes then activated during ionic stress. Other putative TFs related to salt tolerance that were identified in this study included AP2-EREBP, MYB, C2C2-Dof, bZIP, and bHLH (see Additional file 2). Previous research has also demonstrated that some differentially expressed TF genes are associated with plant salt tolerance, including *GhHB2* [68], *GhDREB1A* [76], *AtC3H29* [59], *AtPP2C66* [77], and *OsC3H33* [78]. The discovery in this study of many TFs in various families indicates that a complicated transcriptional regulation network is involved in response to high salinity in cotton.

### Differentially expressed regulators and functional unigenes in tolerant and sensitive genotypes under salt stress

**Figure 11:**
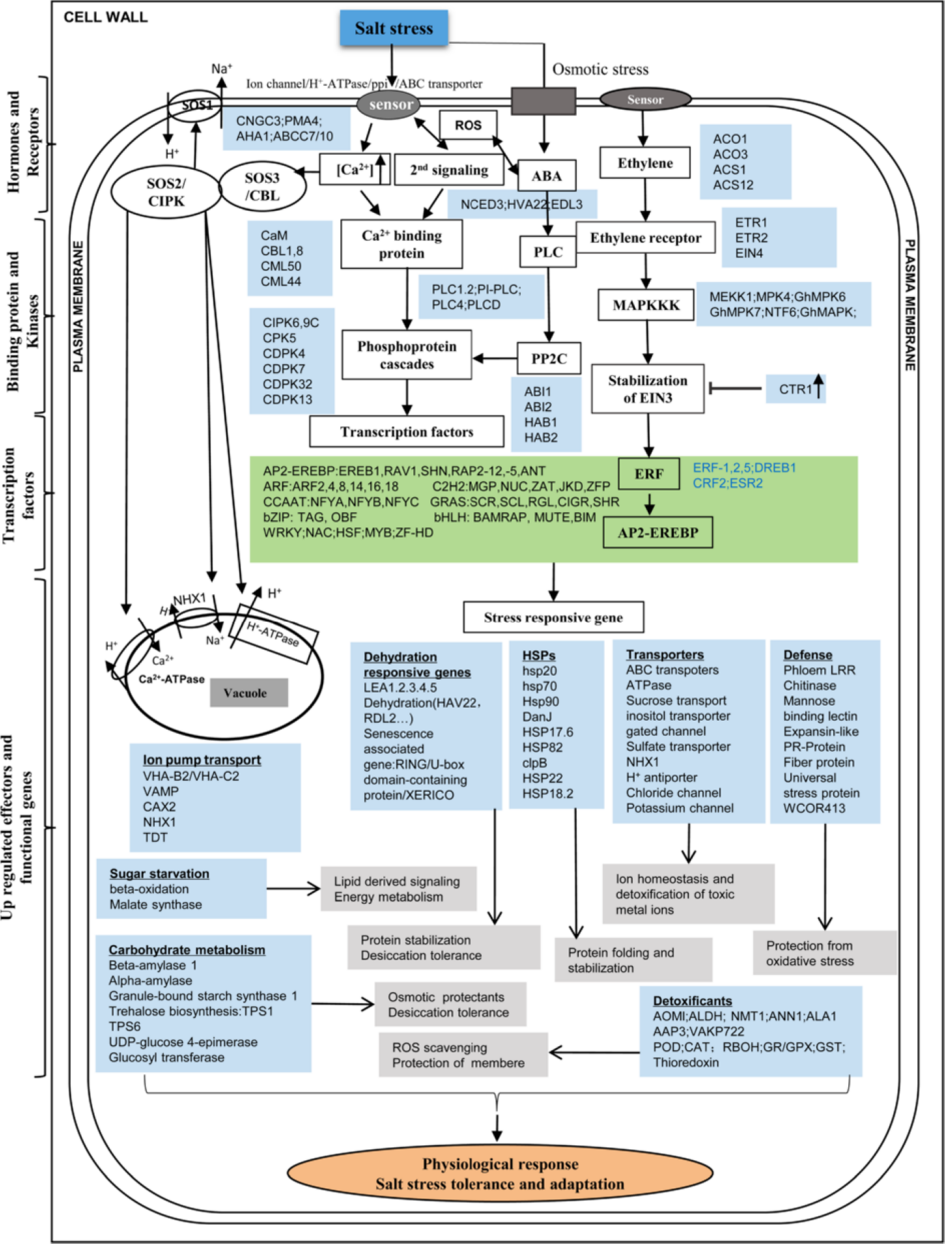
**Overview of genes differentially expressed under salt stress at 4 and 24 h in** ***Gossypium hirsutum*** **.** Up-regulated genes (*wathet blue and green*) expressed at various stages (4 and 24 h) are indicated in the boxes. Receptor, kinase, TF, and effector genes that were differentially expressed under salt stress are listed in Additional file 4.

When a plant experiences sudden salt stress, salinity perception occurs via ionic and osmotic stress signaling. These signals are first sensed by receptors present on plant cell membranes, with a large number of cell-membrane transport pathways, including ion channels and ion carriers, also participating in this signal transduction. Many of these receptors and transporters have been characterized at the gene level, revealing that the uptake and distribution of particular ions may involve some of the genes and gene families identified in our study (see Additional file 4 and Figure 11). Plant cyclic nucleotide-gated ion channels (CNGCs) are mainly involved in developmental processes, plant-pathogen interactions, and initiation of cytosolic Ca^2+^-dependent signal transduction. In our study, *GhCNGC* was identified and differentially expressed under 4- and 24-h salt stress in the salt-tolerant genotype. CNGC3 has only limited involvement in long-term Na^+^ uptake, as revealed by an earlier study that found that short-term uptake of Na^+^ was greater in the *cngc3* mutant relative to the wild type, but uncovered no significant differences in Na^+^ content after prolonged periods [79]. Several other key ABC transporter and H^+^-transporting ATPase genes, such as *ABCB21, ABCC10, ABCG39, AHA1, AHA5,* and *VAMP722*, were significantly up-regulated at 4 and/or 24 h of salt treatment. Extracellular stress signals are first perceived by membrane receptors, which then activate a large and complex intracellular signaling cascade that includes the generation of second messengers such as Ca^2+^. This increased cytosolic Ca^2+^ initiates the stress signaling pathways required for stress tolerance [80].

CDPK, SOS, and MAPK pathways are important signaling pathways associated with salt stress response [81]. CaMs/CMLs, CDPKs, and CIPKs represent the three major classes of plant Ca^2+^ sensors that are involved in many developmental and stress-induced signal transduction pathways [82]. In the present study, CDPKs (homologous to *AtCPK7*and *AtCPK30*) were mainly up-regulated at 4 and 24 h in the salt-tolerant genotype, with their expression remaining at low levels over 24 h of salt stress in the salt-sensitive genotype. We also found that a *CPK5* gene, homologous to *PtCPK5*, was up-regulated at 4 and 24 h. *GhCDPK32*, homologous to *AtCPK32*, was identified and exhibited peak expression at 24 h of treatment (see Additional file 4). *AtCPK32* overexpression has been reported to affect the expression of several ABF4-regulated genes and to be involved in ABA/salt-stress response [83]. Furthermore, a *GhCPK5* gene was cloned and found to be responsive to salt tolerance [84]. Overexpression of *OsCPK7* and *AtCPK30* enhances tolerance to cold and to salinity and drought [85, 86]. The observed up-regulation of *MEKK1-MPK4/6* at 4 and 24 h of salt treatment indicates that the MAPK signaling pathway mediates salt-stress signaling in cotton (Figure 11). In addition, *GhMPK6* was up-regulated under salinity at two time points in the salt-tolerant genotype, while its expression remained unchanged in the salt-sensitive genotype. *GhCAT1* was also over-expressed both at 4 and 24 h in the salt-tolerant genotype (see Additional file 4). The genes in the MAPK signaling pathway likely exist in cotton as well. The role of the MEKK1-MKK2-MPK4/MPK6 cascade in cold- and salt-stress signaling has been demonstrated in Arabidopsis [87]. Luo et al. [88] have suggested that *GhMPK6* plays a major role in ABA-induced CAT1 expression and H_2_O_2_ production. Although GhMPK7 has an important function in salicylic acid (SA)-regulated broad-spectrum resistance to pathogen infection [89], it was a novel MAPK involved in salt-stress response. The expression of genes involved in MAPK and Ca^2+^ signaling pathways, along with other kinases, at various time points under salt stress suggests that the induction of signaling pathways can activate responsive targets for adaptation to salt stress in cotton.

Plant hormones such as ABA, ethylene (ET), SA, and jasmonate (JA) are essential for plant adaptation to abiotic stress conditions [90]. In our study, cotton unigenes associated with ABA and ET signaling pathways showed differential expression at different time points under salt stress (Figure 11 and Additional file 4). Four genes homologous to *AtNCED3, AtHVA22A, AtHVA22E*, and *AtEDL3,* which are related to ABA synthesis or regulation, were up-regulated after 4 and 24 h of salt treatment in the salt-tolerant line. Up-regulation of *PLC2*, *ABI1/2*, and *HAB1/2* at 4 and 24 h, *PLC4* at 4 h, and *PLC1* at 24 h in the salt-tolerant genotype implied that ABA signaling pathway induction occurred under salt stress (Figure 11). As one of the main plant hormones, ABA regulates numerous developmental processes and adaptive stress responses in plants. Recent studies have shown that 9-cisepoxycarotenoid dioxygenase (NCED3) is the key enzyme involved in ABA biosynthesis [91]. *AtHVA22* is differentially regulated by ABA, cold, dehydration, and salt stresses [92]. In *A. thaliana*, strong and rapid induction of the *EDL3* gene has been observed under osmotic stress and high salinity [93]. Phospholipase C (PLC) and protein phosphatase 2C (PP2C) are involved in the ABA signaling pathway [94]. During salt stress (between 4 and 24 h), genes related to ET biosynthesis (homologs of *ACS1*, *ACS12*, *ACO1*, and *ACO3*) were also up-regulated. ET receptor (*ETR1*, *ETR2*, and *EIN4*), ET signaling pathway (EIN3, ERF1, ERF2, and *MEKK1-MKK2-MPK4/6* kinases), and feedback mechanism (CTR1) genes were also up-regulated at 4- and/or 24-h time points (Figure 11 and Additional file 4). ET regulates stress and defense responses along with many key events of plant growth and development. It binds to membrane protein receptors (ETR1, ETR2, ERS1, ERS2, and EIN4) and activates the MAPK cascade (MKK9-MPK3/MPK6) which, in turn, stabilizes the EIN3 protein. EIN3 activates the expression of ERF1 involved in secondary transcription in the signaling pathway, while CTR1 (belonging to MAPKKK) acts as a negative regulator and degrades EIN3 [95]. In our study, only *AtMPK6* was detected. We suspect that additional genes exist that were undetected. An earlier study also revealed that ET is involved in drought-stress tolerance as well as cotton fiber development [96]. Our results suggest the occurrence of multiple-hormone cross-talk in response to osmotic and salt stresses at different time points.

Some salt-responsive downstream genes, especially those involved in antioxidant system and transport functions (ion translocations across plasma and vacuolar membranes), may also provide a well-characterized clue to understanding the mechanisms responsible for salt tolerance. Under salt stress, several transporters of toxic metal ions, lipids, water, and sugar molecules were differentially expressed, such as ABC transporters, H^+^-ATPase, sucrose transport, inositol transporter, gated channel, sulfate transporter, NHX1, H^+^ antiporter, chloride channel, and potassium channel proteins. Another aspect of salt tolerance is the regulation of ROS to protect membranes and macromolecules. In our study, several genes related to detoxification were up-regulated at various time points under salt stress. These expressed genes were homologous to *AOMI, ALDH, NMT1, ANN1, ALA1, AAP3, VAKP722, POD, CAT, RBOH, GR/GPX*, and *GST* [97–99].

### Mediation of a potential regulatory network via conserved miRNAs in the *G. hirsutum*salt-tolerant genotype

Over the past few years, miRNAs have been reported to be prominent gene regulatory factors in plant tolerance to environmental stresses such as drought, cold, heat, and high salinity [100]. Much effort has been devoted to understanding their role in salt-stress responses of various plants including *Zea mays* [39], *Populus euphratica* [101], *Gossypium hirsutum* [40], and *Thellungiella salsuginea* [102].

In our study, salt-responsive miRNAs were detected in both cotton genotypes. A majority of the responsive miRNAs were either detected only in the salt-tolerant genotype or had distinct and inverse expression trends at 4- or 24-h time points between the two cotton genotypes (Figure 8B; Additional file 9A). This dichotomy suggests that common-regulated miRNAs may be part of the fundamental mechanism of salt-stress adaptation, while differentially expressed miRNAs are responsible for the distinct salt sensitivities between the two cotton genotypes (see Additional file 10). Our results are consistent with a previous hypothesis that highly expressed miRNAs are primarily responsible for control of basic cellular and developmental pathways common to most eukaryotes, whereas lower-expressed miRNAs are involved in regulation of lineage-specific pathways and functions [103].

Because miRNAs regulate the specific genes targeting mRNAs for degradation or translation inhibition, identification of the potential target unigenes of miRNAs is crucially important for understanding miRNA-mediated processes such as salt tolerance in plants. According to the PCC and linear regression analysis, the candidate negative correlation miRNA/target genes were further analyzed (Figure 7). It is noted that there is four clumps along the X-axis obviously: miRNA strongly down, slightly down, slightly up, and very strongly up (Figure 7B, E). Since some miRNAs were strongly repressed in salt stress than that in control condition,and some miRNAs were strongly inducted in salt stress compared with that in control condition, the log_2_(E4/E0) and log_2_ (E24/E0) value of some miRNAs in different treatment were more than 10 or less than -10. These miRNAs may be play an important role in response to salt stress. The identified differentially expressed miRNAs which their log_2_ (E4/E0) and log_2_ (E24/E0) value range of -2 ~ -1 and 1 ~ 4 can regard as slightly down and slightly up-regulated miRNAs. Therefore, the data points are separated into four clumps along the X-axis. There is no doubt that all these candidate negative correlation miRNA/target genes should be emphasized. Here, we found that the differentially expressed, conserved miRNA targets of salt-tolerant Earlistaple 7 generally encoded TFs, enzymes, and stress response, transport, and signal transduction proteins (Figure 9).

Many miRNA target genes encode mRNAs of TFs, indicating upstream regulation of miRNAs during developmental processes and environmental responses [104, 105]. Some miRNAs, including miR156, miR164, miR166, miR171, miR172, miR393, miR396, and miR397, exhibited altered expression profiles in at least one genotype in our study. Based on transcriptome data prediction results, they regulate the expression of TFs (Figure 9). In this study, three miR156s (bna-miR156a, osa-miR156f-3p, and gma-miR156q) and one miR172 (zma-miR172a) were up-regulated and down-regulated, respectively, under 24-h salt treatment in the tolerant line Earlistaple 7. In addition, three of the above miRNAs may function by changing normal levels of downstream gene transcripts through modulation of SPL5 and RAP2-7 expression (see Additional file 11). miR156 and miR172 are also involved in the regulation of flowering time and floral development in Arabidopsis, which they accomplish by negatively regulating SBP-LIKE proteins (SPLs) and AP2-like factors, respectively [106]. These results suggest that the three conserved miRNAs may play significant regulatory roles in shoot maturation and the vegetative- to reproductive-phase transition. The NAC family is involved in embryo and shoot meristem development, lateral root formation, auxin signaling, and defense and abiotic stress responses [107]. The conserved miRNA ptc-miR164f, which was verified by stem-loop RT-PCR to target the NAC domain-containing protein (*NAC021/AtNAC1*), was highly down-regulated at 4 and 24 h of salt stress in Earlistaple 7 (see Additional file 11 and Figure 10B). We observed that the CUP-SHAPED COTYLEDON 2 gene (comp81541_c0_seq1; homologous to *AtNAC098*), which is targeted and negatively regulated by at-miR164a, was especially down-regulated only at 24 h in Earlistaple 7 (see Additional file 11 and Figure 9). This result suggests that the different miRNA164s may negatively regulate different NACs during at the early stage of salt stress of salt stress. The No Apical Meristem (NAC) protein is involved in plant hormonal control and defense [108, 109]. In Arabidopsis, *AtNAC1*-overexpressing lines are bigger than the wild type, with larger leaves, thicker stems, and more abundant roots [108], suggesting that NAC1 may be an early auxin-responsive gene. CUP-SHAPED COTYLEDON 2 (a NAC family member) and at-miR164A are transcribed in overlapping domains at the margins of young leaf primordia, with transcription gradually restricted to the sinus where the Arabidopsis leaf margins become serrated [110]. Most often, the regulatory pathway consisting of miR393 that targets TIR1, a negative regulator in auxin signaling, may participate in lateral root development in Arabidopsis [111]. The other target was a bHLH transcription factor, which was involved in many biological processes, but there were no phenotypic changes and functional mechanisms owing to miR393/bHLH interaction in Arabidopsis [112]. In our study, all miR393s (ath-miR393a, osa-miR393a, csi-miR393, and osa-miR393b-5p), which were highly expressed at 4 and 24 h of salt stress in salt-tolerant Earlistaple 7 but were unaffected or down-regulated in salt-sensitive Nan Dan Ba Di Da Hua, targeted and tested as negatively related to a bHLH78 homolog unigene (comp105131_c0_seq1) by stem-loop RT-PCR (see Additional file 11 and Figure 10B). Induction of miR393 at the early stage of salt stress (4 and 24 h in the salt-tolerant genotype) suppressed the expression of its target *A*t*bHLH78*.In our proposed miRNA-mediated regulatory network based on miRNA-TF interactions (Figure 9), one miRNA may regulated more than one TF gene. In addition, differentially expressed conserved miRNAs may also target non-TF genes. Comparison of target genes of conserved miRNAs illustrated in the regulatory network suggests that the same miRNA response to salt stress at different time points may regulate the same or different genes to exert different functions. Nevertheless, these putative interactions need to be confirmed by experimental data.

## Conclusions

In this study, we first generated a global transcription map of genes and miRNAs expressed in leaves of salt-tolerant cotton under salt stress. We detected 29,136 and 33,492 unigenes exhibiting differential co-expression after 4 and 24 h of salt stress, respectively. GO annotations demonstrated that unigenes belonging to a wide range of functional categories are involved in salinity defense response at the two different time points. We also identified 320 miRNAs conserved across the two genotypes. Of these, 108 members of miR156, miR159, miR164, miR167, miR171, miR172, miR393, miR396, miR397, and miR482 families were differentially expressed at 4 and 24 h under salt stress in the salt-tolerant genotype. Some miRNA targets were predicted to encode abiotic stress-responsive TFs and enzymes. Expressions of some selected differentially regulated miRNAs and their targets were found to be negatively regulated. Comparative transcriptome analysis of salt-tolerant and salt-sensitive genotypes indicated that essential salt tolerance-related genes and miRNAs were differentially expressed and were only weakly or non-responsive to salt stress in the salt-sensitive cotton. Finally, we have provided a list of salt tolerance-related genes that will facilitate candidate gene discovery and molecular marker development for salt-tolerant breeding in cotton.

## Methods

### Plant materials and NaCl treatments

Plant materials comprised two upland cotton (*G. hirsutum*) genotypes: salt-tolerant Earlistaple 7, which was selected from Earlistaple 808 and introduced by the AR-SEA-USDA Pee Dee Experiment Station in the United States in 1982, and salt-sensitive Nan Dan Ba Di Da Hua, introduced from Guangxi Province, China, before 1978. Seeds of the two cotton genotypes were available in the National Mid-term Genebank of the Institute of Cotton Research, Chinese Academy of Agricultural Sciences (ICR-CAAS) after signing the Material Transfer Agreement (MTA) [113]. Seedling cultivation and all experiments were carried out at the State Key Laboratory of Cotton Biology, Anyang, China.

Hand-selected seeds (120 of each genotype) of Nan Dan Ba Di Da Hua and Earlistaple 7 were surface-sterilized in 70% (v/v) ethanol for 15 s and 4% (w/v) sodium hypochlorite for 15 min, and then rinsed several times with sterile distilled water. The seeds were submerged in sterile water for 12 h at room temperature and then sown in sterile silica sand. After 3 days, germinated seedlings were transferred to hydroponic containers (600 × 350 × 120 mm) containing half-strength Hoagland’s solution (pH 6.0) [114] and grown in a phytotron (KR-III; Zhengzhou Henan Ke-rui Inc.). Growth conditions were 28/22 day/night temperature, 60–80% relative humidity, and a 14 h/10 h light/dark cycle under 450 μmol⋅m^-2^⋅s light intensity. The culture solutions were changed after about 14 days, when seedlings had produced three leaves. Seedlings showing normal growth were randomly divided into two groups; one group was placed into tanks filled with a 200 mM solution of NaCl, and the remaining seedlings were transferred to tanks filled with plain water to serve as control. After exposure to the two solutions for 4, 24, 48, and 72 h, seedlings of control and treated Nan Dan Ba Di Da Hua and Earlistaple 7 were harvested directly into liquid nitrogen and stored at -80°C until used for RNA extraction.

### Leaf RWC, REC, chlorophyll content, and ion measurements

Plant fresh weight (FW) was measured immediately after harvest. The leaves were floated on deionized water for 8 h at 4°C. The turgid leaves were quickly weighed (TW) and their dry mass (DW) was measured after oven-drying at 105°C for 10 min followed by 80°C for 24 h. RWC was calculated as follows: RWC (%) = (FW - DW) / (TW - DW) × 100 [115]. For REC, 0.5 g of fresh leaves were rinsed 3 times with ddH_2_O and were placed in an Erlenmeyer flask containing 40 mL of ddH_2_O incubated at room temperature for 4 h. The electrical conductivity of the solution (C1) was measured using an EM38 conductivity meter (ICT international, Armidale, NSW, Australia). The solution was boiled for 10 min and cooled to room temperature, and electrical conductivity (C2) was re-measured. REC was calculated as C1 / C2 × 100% [116]. To measure leaf chlorophyll, 0.2 g of seedling leaves were incubated in 80% acetone in darkness at 4°C overnight. After centrifugation at 5000 × *g* for 5 min at 4°C, the absorbance of the supernatant was measured at 663 and 645 nm with a DU800 spectrophotometer (Beckman Coulter, Brea, CA, USA). Chlorophyll content was calculated as described by Porra (1989) [117]. Concentrations of Na^+^ and K^+^ in roots and leaves were determined by inductively coupled plasma-optical emission spectrometry (ICP-OES) (Optima 2100 DV; Perkin-Elmer, Wellesley USA) according to the manufacturer’s instructions. Fifteen shoots per genotype were analyzed (3 from control and 12 from salt-treated plants). To ensure more accurate measurements, five independent plants were pooled as a sample (about 0.2–0.6 g) to reduce the effects of biological variation. For the Na^+^ and K^+^ determinations, the samples were completely digested in 12 mL 65% HNO_3_ and then analyzed on the ICP-OES spectrometer against National Institute of Standards and Technology traceable standards and controls. For the physiological experiments, three or more independent biological replicates of each control and time-treated sample were performed.

### RNA extraction and qRT-PCR

Total RNA was isolated from cotton leaf samples treated with either 200 mM NaCl or water (as a control) for 4 or 24 h using a modified CTAB method and purified using Qiagen RNeasy columns (Qiagen, Hilden, Germany). First-strand cDNA was synthesized using a PrimeScript RT reagent kit with gDNA eraser (Takara, Dalian, China) according to the manufacturer’s instructions. Primers are listed in Additional file 5A. qRT-PCR was performed on a 7500 Fast Real-Time PCR system (Applied Biosystems, Inc., California USA). The 20-μL reaction solutions contained 10 μL SYBR Green Premix Ex *Taq* (Takara), 1 ng cDNA sample, and 0.2 μM gene-specific primers. Three replicate PCRs were run per cDNA sample, and three cDNAs from different samples were used as biological replicates. Relative expression levels were calculated by the 2^-ΔΔT^ method [118] using *GhActin* (GI: AY305733) as an internal standard to normalize cDNA content [119].

The expression profiles of 10 salt-responsive mature miRNAs were assayed by stem-loop RT-PCR. Stem-loop reverse-transcription primers were designed following the method described by Chen et al. [120] and Varkonyi-Gasic et al. [121]. Their 3′ ends, which were complementary to the 6-nt 3′ end of the miRNA, were combined with the 44-nt sequence 5′-GTCGTATCCAGTGCAGGGTCCGAGGTATTCGCACTGGATACGAC-3′ (Additional file 5B) to constitute the primer. Subsequent PCRs used a 5′ primer matching the ~18 nt at the 5′ end of the target miRNA. The 3′ primer was a universal reverse-transcription primer. The stem-loop reverse transcription reactions were performed using a PrimeScript RT reagent kit with gDNA eraser (Takara) according to the supplier’s manual. U6 snRNA was used as a reference for miRNA expression validation. Stem-loop RT primers and miRNA-specific PCR primers are listed in Additional file 5B, C. As in the qRT-PCR analysis, the 2^-ΔΔT^ method was used to calculate relative expression levels of salt-responsive mature miRNAs.

### Transcriptome sequencing and *de novo*assembly

Poly(A) mRNA was isolated from 10 μg total RNA of each leaf sample using oligo (dT) beads. The mRNA was sheared into short fragments with fragmentation buffer and selected for construction of Illumina RNA-Seq libraries according to the described protocol. Each library had an insert size of approximately 200 bp and was sequenced on a HiSeq 2000 system (Illumina, California USA). Leaf-sample read lengths were 90 bp. The raw image data was transformed by base-calling into sequence data and stored in FASTQ format. To control for the influence of error rate in the Solexa data results, quality pretreatment of the raw data was performed: the sliding window method was first used to remove low-quality fragments (quality threshold = 20, error rate = 1%, window size = 5 bp, and length threshold = 35 bp), followed by removal of partial (<35 bp) sequences containing “N” from the reads. After filtering of low-quality and dirty raw reads, transcriptome *de novo* assembly was carried out using the paired-end assembly method as implemented in Trinity (http://sourceforge.net/projects/trinityrnaseq/files/; trinityrnaseq_r2012-10-05) [122]. After removal of repeats from the spliced sequences, transcripts were obtained of the expected length and size. The longest transcript in each locus (“comp * _ * _c”; Chrysalis Clusters module) was taken as the unigene. To assign possible annotations, all unigene sequences were searched using Blastx (*e*-value < 10^-5^) against the following protein databases: NR (in NCBI), SWISS-PROT (UniProt), TrEMBL (http://www.bioinfo.pte.hu/more/TrEMBL.htm), CDD (in NCBI http://www.ncbi.nlm.nih.gov/cdd/), PFAM (http://pfam.janelia.org/), and KOG (http://www.ncbi.nlm.nih.gov/COG/). GO functional annotations were obtained using the Blast2GO program [123], and all unigenes were assigned into GO functional categories of molecular function, biological process, and cellular components using WEGO software [124]. To identify possible TFs in the transcripts, we used the BLAST tool, sequence information in the PlantTFDB database (http://planttfdb.cbi.pku.edu.cn), and annotations from the SWISS-PROT and NR databases.

### Identification and functional analysis of DEUs

To identify genes displaying significant expression changes during NaCl treatment, differentially expressed transcripts were analyzed by comparing 4- and 24-h libraries with the control library, with the number of reads in all six libraries normalized to reads per kilobase of exon model per million mapped reads (RPKM) [125]. For better accuracy, only unigenes longer than 200 bp were included in the differential unigene expression analysis. Using Bowtie v0.12.8 software and the single-end mapping method, each read per unigene was counted as 1/n by permitting alignment of one read on multiple unigenes. RPKM were calculated according to the formula RPKM = 10^6^ × C / 10^-3^ × N × L, where C is the number of reads uniquely aligned to the unigene, N is the total number of reads uniquely aligned to all unigenes in the specific sample, and L is the number of nt in the unigene. The *P*-value corresponding to a differentially expressed unigene in two samples was determined from Audic’s algorithm [126], with the FDR method applied to determine the *P*-value threshold in multiple tests. To determine whether a unigene was differentially expressed between two samples, we therefore used threshold criteria based on the FDR statistical method and the ratio of RPKMs, i.e., FDR < 0.001 and |log_2_ Ratio| ≥ 1.

Gene expression patterns uncovered during the investigated two time points were analyzed and interpreted using STEM v1.3.8 [127]. GO enrichment analyses for differentially co-expressed unigenes were performed with the differential and full unigene sets treated as prospect and background, respectively, using the hypergeometric distribution algorithm “phyper” in STEM. *P-*values (<0.05 or 0.01) were calculated between the prospective unigene and selected GO subcategories, followed by FDR adjustment.

### Small RNA sequencing and identification of conserved miRNAs

For small RNA library construction, total RNA was separately extracted from the leaves of the same six samples used for generation of mRNA-seq libraries. After confirming RNA quality by agarose gel electrophoresis (28S:18S > 1.5) and on an Agilent 2100 bioanalyzer (RNA Integrity Number ≥ 8.0), the extracted RNA was separated by 15% denaturing polyacrylamide gel electrophoresis (PAGE) to recover the population of small RNAs (size range 17–35 nt) present. The small RNAs were ligated at their 5′ and 3′ ends to a pair of Solexa adapters, and the resulting ligation products were gel-purified by 10% denaturing PAGE and reverse transcribed. The cDNAs obtained in this fashion were sequenced on an Illumina HiSeq 2000 system (Illumina, California USA). After removal of low-quality reads and adapter sequences from the Solexa sequencing reads, unique sequences of 17–35 nt were used for further analysis. First, the unique sequences were queried against ribosomal and transfer RNAs from tRNAdb (http://trna.bioinf.uni-leipzig.de/DataOutput/Search), SILVArRNA (http://www.arb-silva.de/), and NONCODE v3.0 (http://www.noncode.org/NONCODERv3/; plant database only, single-nt mismatches allowed) databases to obtain matching rRNA, tRNA, snRNA, and snoRNA sequences. Any small RNA sequences having exact matches to these sequences were removed from the analysis. The remaining unique small RNA sequences were then BLASTn-searched against the conserved plant miRNAs in the miRBase database (miRBase 19.0; http://www.mirbase.org/) to identify conserved miRNAs. Only perfectly matched sequences were considered to be conserved miRNAs. Because we were only conducting a combined analysis of transcriptome and conserved miRNA data, we did not perform a novel miRNA prediction on the content.

### Analyses of conserved miRNA differential expression, target unigene prediction, and miRNA-target pair functional regulatory relationships

A high-throughput sequencing abundance profile analysis was performed based on the sequence reads in each library. miRNA expression abundance in each library was calculated as RPM (reads per million) according to the formula RPM = mapped reads × 10^6^ / total reads. Calculation of *P-*values for comparing the miRNA expression between salt-treated samples and the control sample was based on previously established methods [126, 128]. Specifically, we used the following log_2_ Ratio formula: log_2_ Ratio = log_2_ (miRNA reads in the salt treatment / miRNA reads in the control). Differential expression of miRNAs in the comparisons N4/N0 (where N is Nan Dan Ba Di Da Hua and E is Earlistaple 7), N24/N0, E4/E0, and E24/E0 were screened based on the chi-square test with filters of *P* < 0.05 and |log_2_ Ratio| ≥ 1.0.

To search target genes, the conserved miRNA sequences were matched against the cotton transcriptome unigene database using psRNATarget (http://plantgrn.noble.org/psRNATarget/). Sequences with less than 3 nt mismatches compared with the query miRNA sequences were selected.

### Statistical analysis

Each data point represented the mean of three or more replicated treatments, with each treatment consisting of at least five plants. Physiological data statistical analyses were performed by Tukey’s method of two-way ANOVA using IBM SPSS Statistics v19.0 (SPSS Inc., Chicago, IL, USA). *P-*values less than 0.05 were considered as statistically significant. R software (http://www.r-project.org) was used for the calculation of Pearson correlation coefficients between miRNAs and their targets and for the construction of a heatmap of co-expressed TF unigenes and miRNA expression profiles.

## Electronic supplementary material

Additional file 1: **Summary of** ***de novo*** **assembled mRNA-seq transcriptome data.** (A) Mapping of the length distribution of all unigenes; (B) GC content of all unigenes; (C) Functional classifications of GO-annotated unigenes. (PDF 238 KB)

Additional file 2: **The characteristic of salt-responsive transcription factor (TF) unigenes in different comparisons.** Eight charts are included. (A) Annotation of 3,172 TF unigenes from all samples; (B) Numbers of differentially expressed unigenes (up and down-regulation) in each transcription factor family for four comparisons; (C) TF unigenes commonly differentially expressed in N-E only at 4 h; (D) TF unigenes commonly differentially expressed in N-E only at 24 h; (E) TF unigenes commonly differentially expressed in N-E both at 4 and 24 h; (F) TF unigenes differentially expressed only in E at 4 h; (G) TF unigenes differentially expressed only in E at 24 h; (H) TF unigenes differentially expressed only in E both at 4 and 24 h. (XLSX 739 KB)

Additional file 3: **Flowchart analysis of all differentially co-expressed unigenes.** (PDF 8 KB)

Additional file 4: **Differentially expressed unigenes at 4- and 24-h of salt stress in Earlistaple 7 (also shown in Figure** 11 **).** (XLSX 231 KB)

Additional file 5: **Specific primer information of unigenes (including 9 miRNA targets) for qRT-PCR and 9 conserved miRNAs for stem-loop RT-PCR.** (A) 26 pairs of transcript-specific primers for qRT-PCR; (B) 9 miRNA primers for stem-loop RT-PCR; (C) 9 miRNA gene primers for real time PCR analysis; (D) 26 represented unigenes QRT-PCR data and mRNA-seq data in four comparisons. (XLSX 19 KB)

Additional file 6: **Summary of small RNA sequencing data.** (A) Statistical summary of data before and after quality pretreatment. (B) Size distribution of small RNA unique reads; (C) Number of detected members of each miRNA family; (D) Abundance of conserved miRNA families. (PDF 168 KB)

Additional file 7: **RPM values of (A) all identified and (B)** ***Gossypium hirsutum*** **conserved miRNAs in six libraries.** (XLSX 52 KB)

Additional file 8: **Differentially expressed conserved miRNAs at 4- and 24-h of salt stress in Nan Dan Ba Di Da Hua and Earlistaple 7.** (XLSX 71 KB)

Additional file 9: **Venn diagram illustrating the numbers of differently up- and down-regulated miRNAs between Nan Dan Ba Di Da Hua and Earlistaple 7 in response to salt stress at (A) 4 h and (B) 24 h; (C) Flowchart analysis of differentially expressed miRNAs and combined analysis with their target genes; (D) Procedure for screening of negative miRNA-target unigene relationships related to salt-treatment response; (E) Gene Ontology analysis of conserved miRNA targets identified in salt-tolerant Earlistaple 7 at 4 and 24 h of salt treatment.** (PDF 572 KB)

Additional file 10: **Detailed information for potential targets of conserved miRNAs at 4- and 24-h of salt stress in Earlistaple 7.** (A) Negatively correlated miRNA-target pairs in Earlistaple 7 at 4-h; (B) Positively correlated miRNA-target pairs in Earlistaple 7 at 4-h; (C) Negatively correlated miRNA-target pairs in Earlistaple 7 at 24-h; (D) Positively correlated miRNA-target pairs in Earlistaple 7 at 24-h. (XLSX 136 KB)

Additional file 11: **Detailed information on differentially expressed conserved miRNAs in different comparisons.** (A) RPM values of 108 differentially expressed conserved miRNAs at 4- or 24-h time points in five comparisons; (B) The genotype-specific salt-responsive conserved miRNAs at 4- or 24-h time points of salt-stress in five comparisons; (C) Seventy-five negatively correlated miRNA-target pairs in salt-stressed Earlistaple 7 at 4- and 24-h time points; (D) Matching of 75 negatively correlated miRNA-target pairs; (E) The salt-responsive differentially expressed conserved miRNAs and their target genes at different treatment time points (relationship shown in Figure 9). (XLSX 76 KB)

## Abbreviations

N: Nan Dan Ba Di Da Hua
E: Earlistaple 7
RWC: Relative water content
REC: Relative electric conductivity
GO: Gene ontology
DEU: Differentially expressed unigene
FDR: False discovery rate
RPKM: Reads per kilobase of gene model per million mapped reads
RPM: Reads per million
qRT-PCR: Real-time quantitative RT-PCR
SLRT-PCR: Stem-loop reverse transcription-PCR
STEM: Short time-servies expression miner
TF: Transcription factor
PCC: Pearson correlation coefficient.
