# Supplementary material for: Comprehensive analysis of differentially expressed genes and transcriptional regulation induced by salt stress in two contrasting cotton genotypes
Source: BMC Genomics. 2014 Sep 5;15(1):760. doi: 10.1186/1471-2164-15-760 (PMC4169805; doi:10.1186/1471-2164-15-760)
Supplement: Supplementary file 1 — Additional file 1: Summary of de novo assembled mRNA-seq transcriptome data. (A) Mapping of the length distribution of all unigenes; (B) GC content of all unigenes; (C) Functional classifications of GO-annotated unigenes. (PDF 238 KB) [file 12864_2014_6456_MOESM1_ESM.pdf]

Additional file 1. Summary of *de novo* assembled mRNA-seq transcriptome data

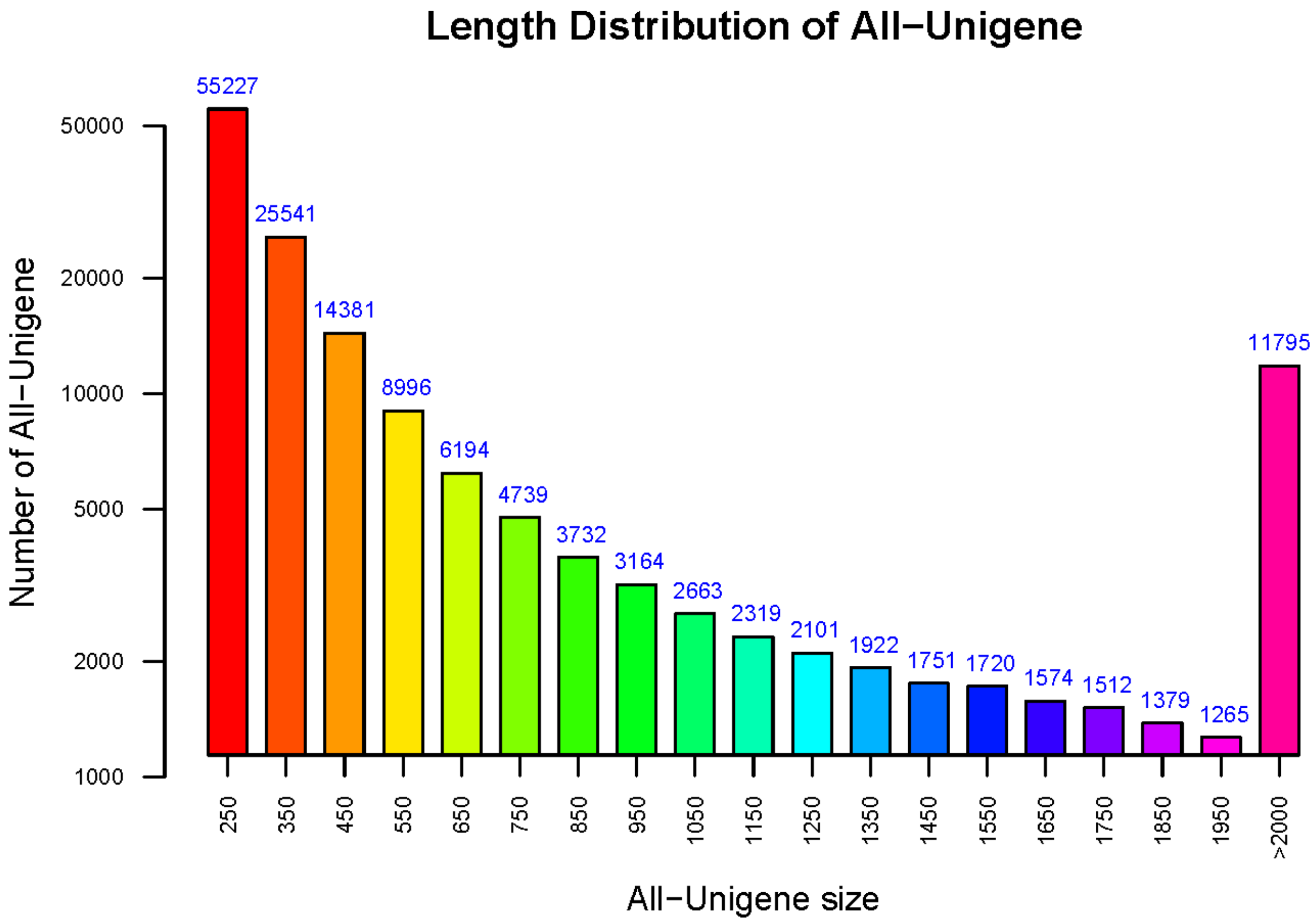

A. Mapping of the length distribution of all-unigene

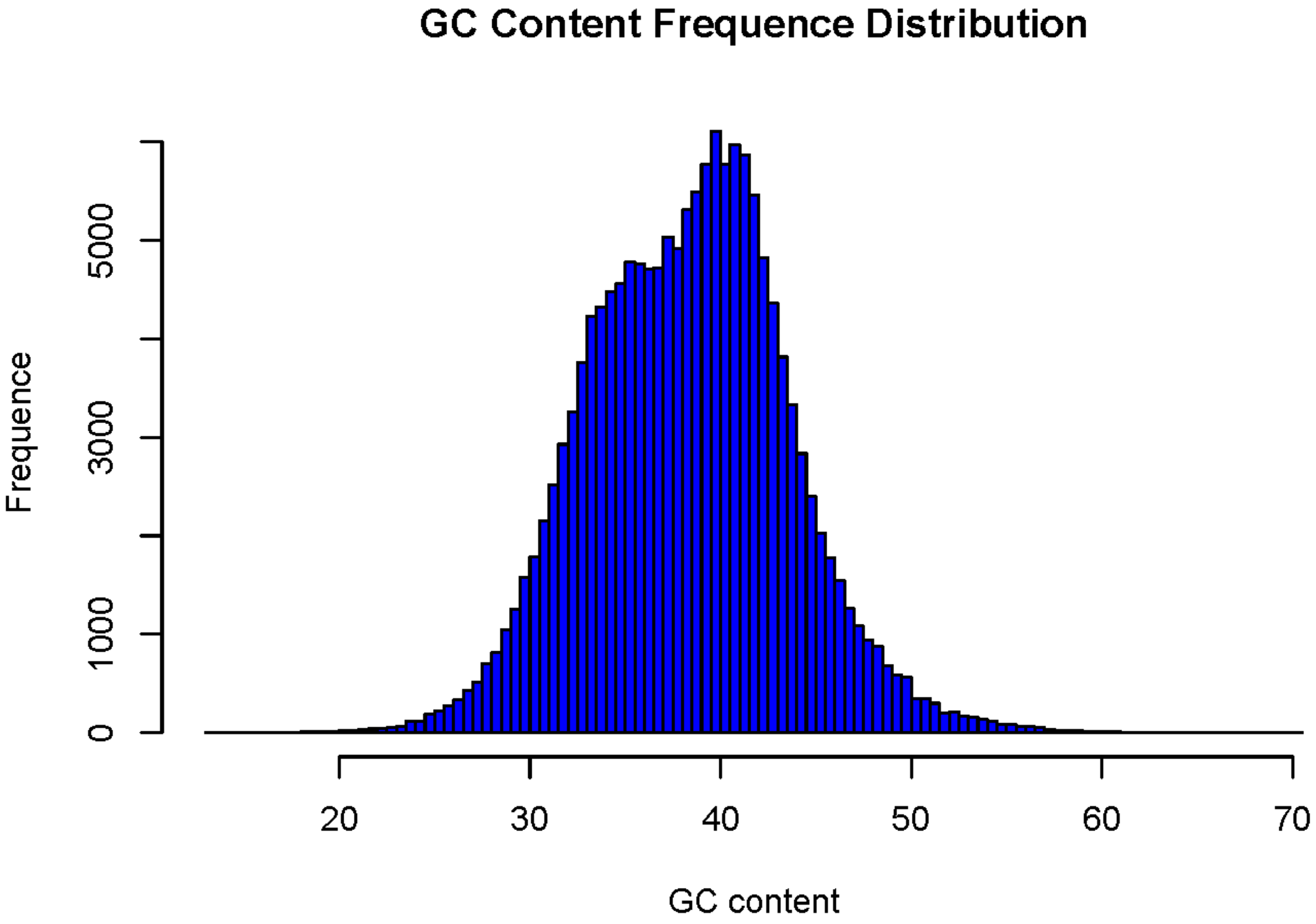

B. GC content of all unigenes

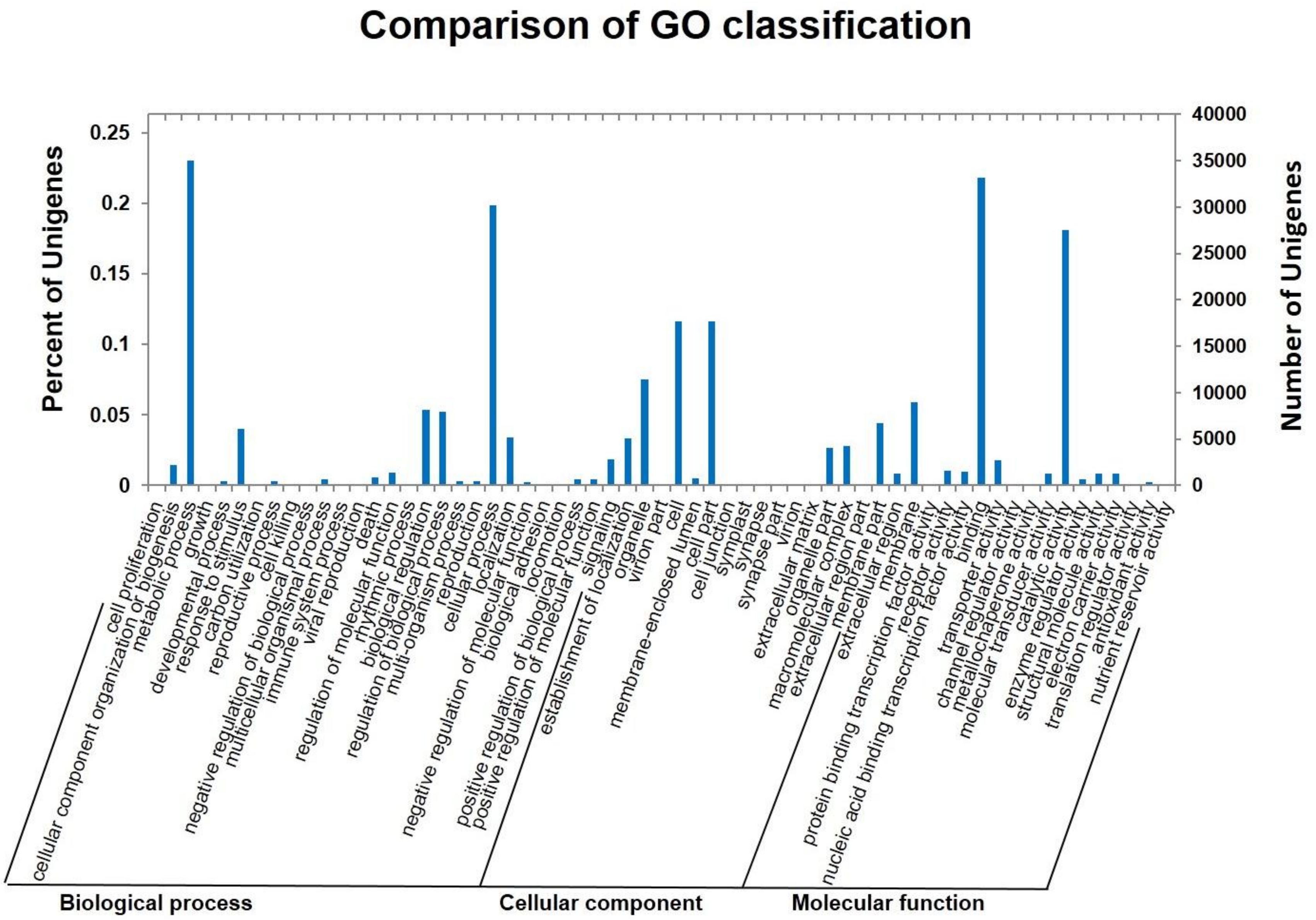

C. Functional classifications of GO annotated unigenes
