# Supplementary figures and images for: Comprehensive analysis of differentially expressed genes and transcriptional regulation induced by salt stress in two contrasting cotton genotypes
Source: BMC Genomics. 2014 Sep 5;15(1):760. doi: 10.1186/1471-2164-15-760 (PMC4169805; doi:10.1186/1471-2164-15-760)

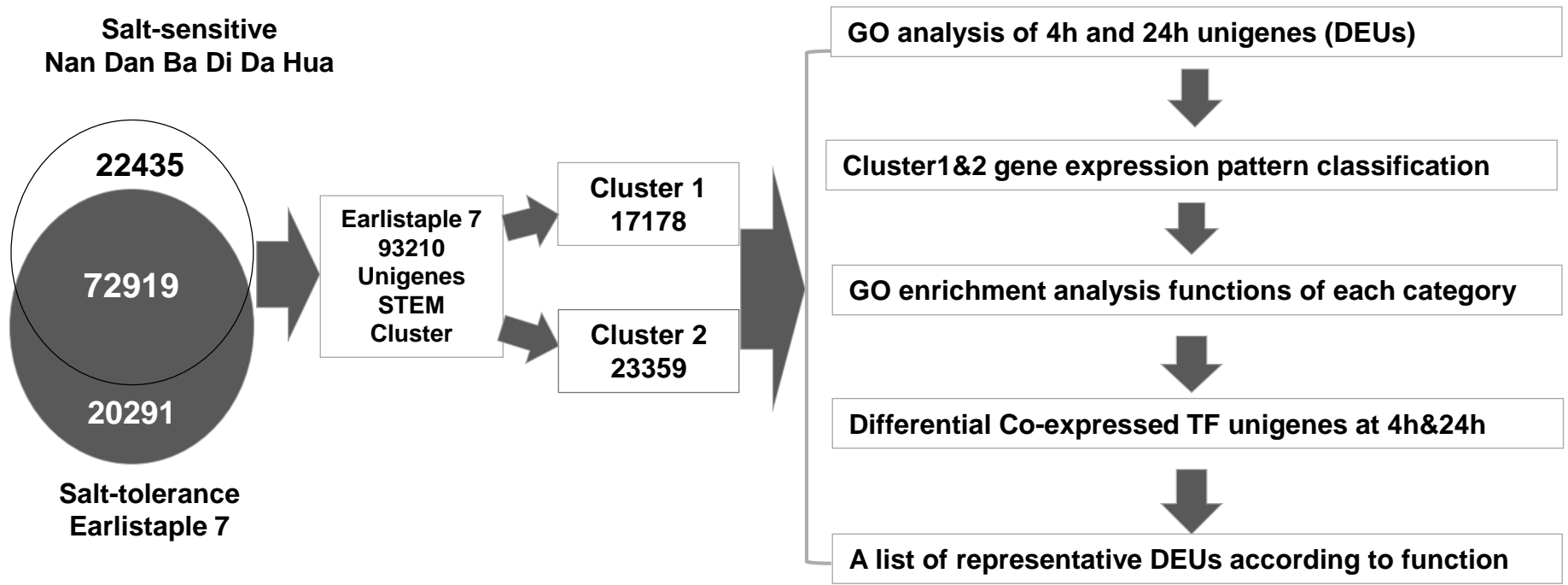

**Additional file 3. Flowchart analysis of the differentially co-expressed unigenes**

Supplement: Supplementary file 3 — Additional file 3: Flowchart analysis of all differentially co-expressed unigenes. (PDF 8 KB) [file 12864_2014_6456_MOESM3_ESM.pdf]
