# Supplementary material for: Comprehensive analysis of differentially expressed genes and transcriptional regulation induced by salt stress in two contrasting cotton genotypes
Source: BMC Genomics. 2014 Sep 5;15(1):760. doi: 10.1186/1471-2164-15-760 (PMC4169805; doi:10.1186/1471-2164-15-760)
Supplement: Supplementary file 6 — Additional file 6: Summary of small RNA sequencing data. (A) Statistical summary of data before and after quality pretreatment. (B) Size distribution of small RNA unique reads; (C) Number of detected members of each miRNA family; (D) Abundance of conserved miRNA families. (PDF 168 KB) [file 12864_2014_6456_MOESM6_ESM.pdf]

Additional file 6. Summary of small RNA sequencing data

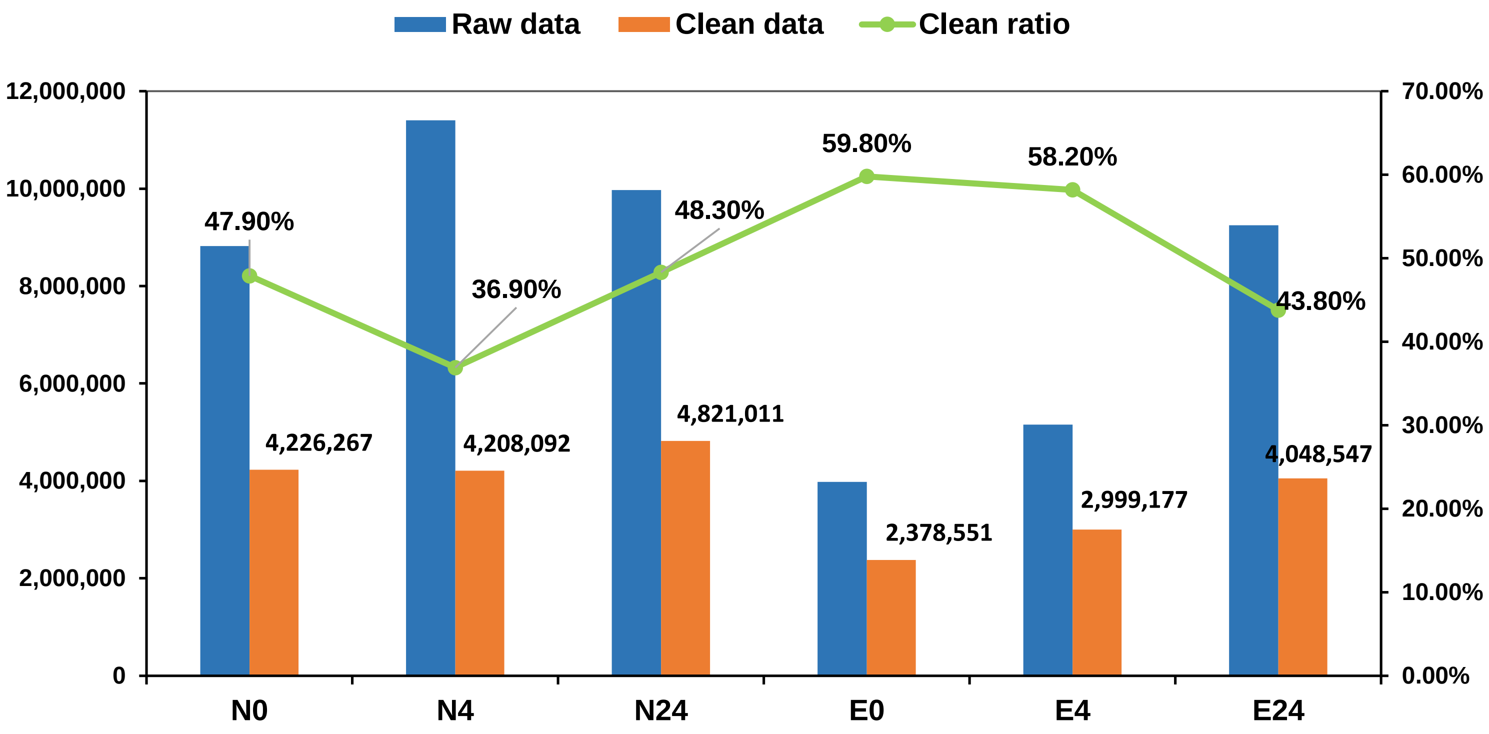

A. Statistical summary of data before and after quality pretreatment

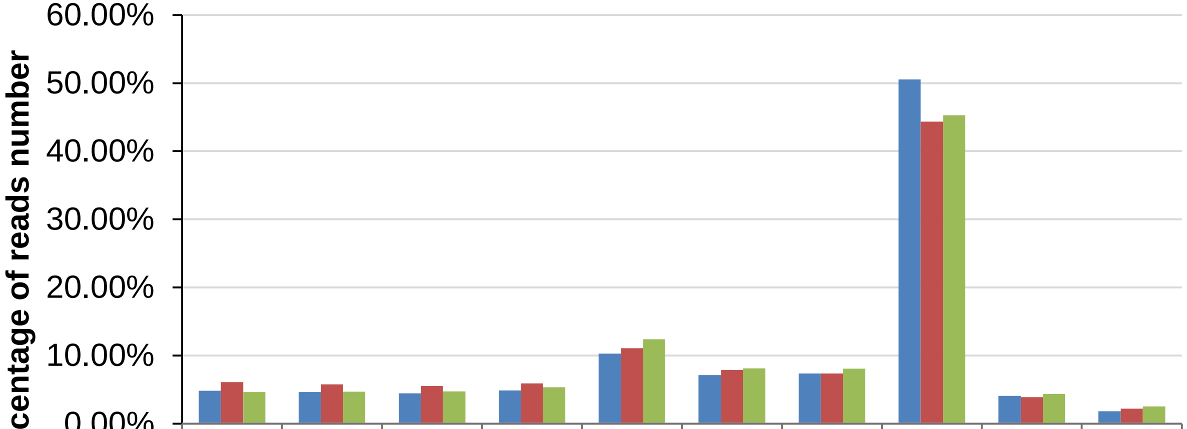

	17	18	19	20	21	22	23	24	25	26
N0	4.81%	4.63%	4.47%	4.88%	10.27	7.14%	7.37%	50.56	4.06%	1.83%
N4	6.09%	5.76%	5.52%	5.90%	11.05	7.85%	7.37%	44.36	3.90%	2.20%
N24	4.62%	4.67%	4.71%	5.32%	12.37	8.12%	8.06%	45.27	4.35%	2.50%

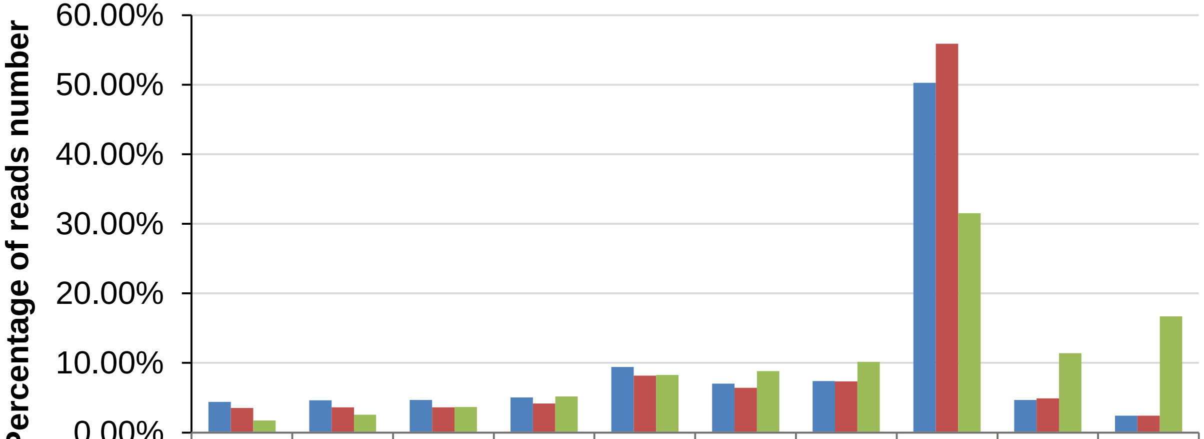

	17	18	19	20	21	22	23	24	25	26
E0	4.41%	4.63%	4.65%	5.04%	9.43%	7.04%	7.41%	50.28	4.70%	2.41%
E4	3.52%	3.61%	3.62%	4.15%	8.16%	6.41%	7.36%	55.87	4.89%	2.40%
E24	1.73%	2.57%	3.67%	5.19%	8.25%	8.81%	10.14	31.55	11.40	16.70

sRNA Length (nt)

B. Size distribution of small RNA unique reads (N: above; E: below)

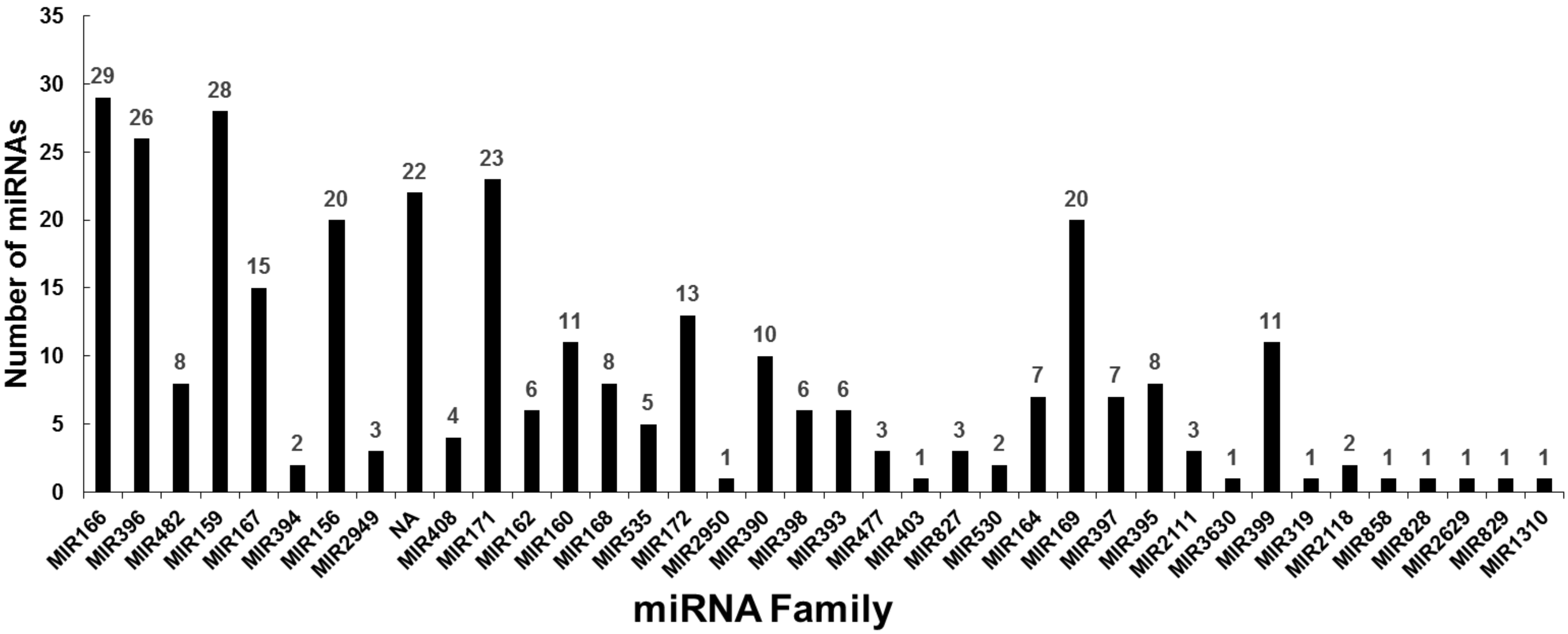

C. Number of detected family members of each miRNA family

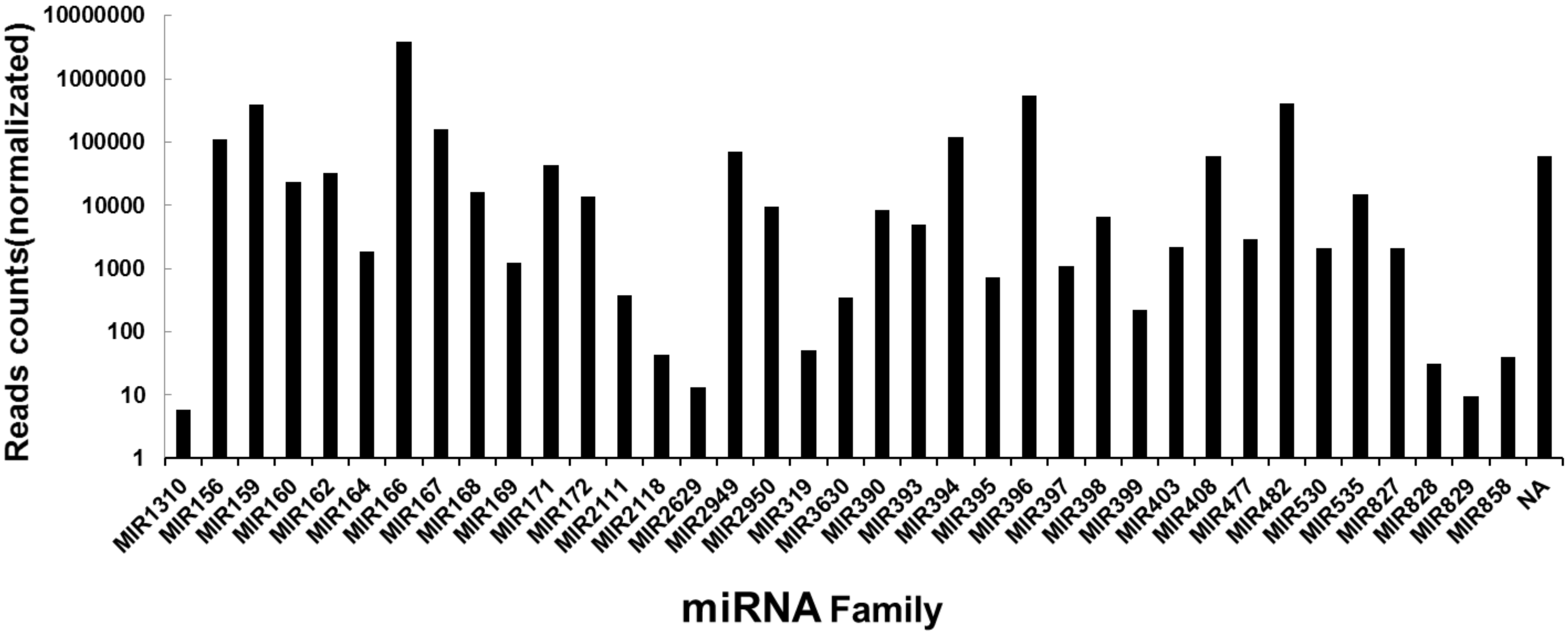

D. Abundance of conserved miRNA families
