# Supplementary material for: Comprehensive analysis of differentially expressed genes and transcriptional regulation induced by salt stress in two contrasting cotton genotypes
Source: BMC Genomics. 2014 Sep 5;15(1):760. doi: 10.1186/1471-2164-15-760 (PMC4169805; doi:10.1186/1471-2164-15-760)
Supplement: Supplementary file 9 — Additional file 9: Venn diagram illustrating the numbers of differently up- and down-regulated miRNAs between Nan Dan Ba Di Da Hua and Earlistaple 7 in response to salt stress at (A) 4 h and (B) 24 h; (C) Flowchart analysis of differentially expressed miRNAs and combined analysis with their target genes; (D) Procedure for screening of negative miRNA-target unigene relationships related to salt-treatment response; (E) Gene Ontology analysis of conserved miRNA targets identified in salt-tolerant Earlistaple 7 at 4 and 24 h of salt treatment. (PDF 572 KB) [file 12864_2014_6456_MOESM9_ESM.pdf]

Additional file 9

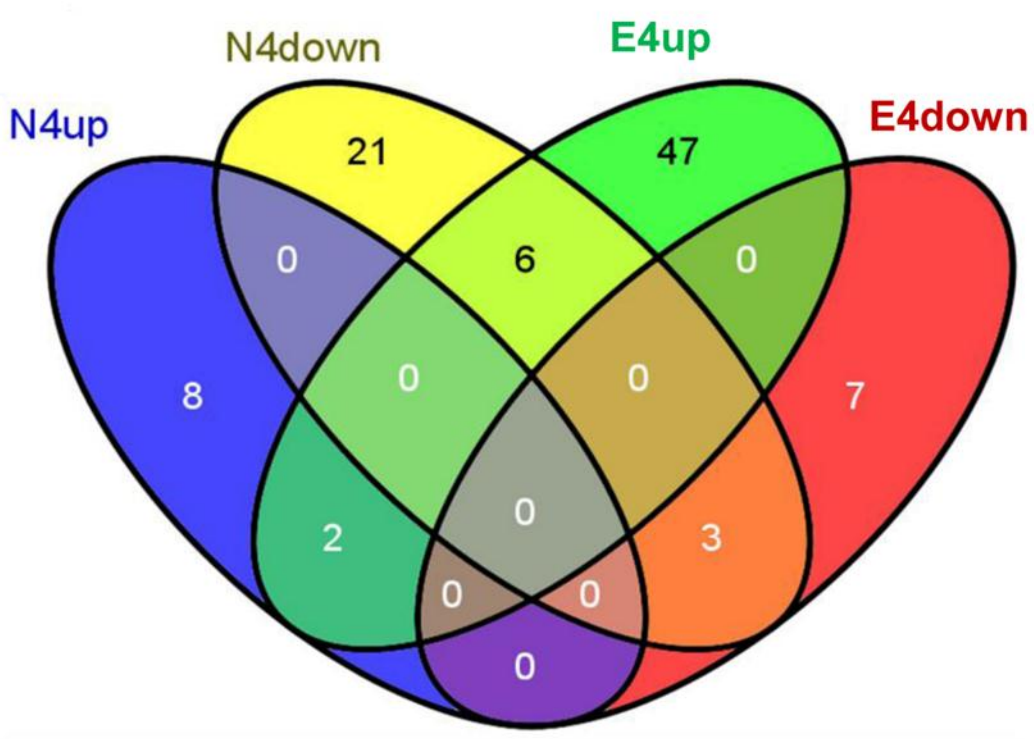

A. Venn diagram illustrating the numbers of differently up- and down-regulated miRNAs between Nan Dan Ba Di Da Hua and Earlistaple 7 in response to salt stress at 4h

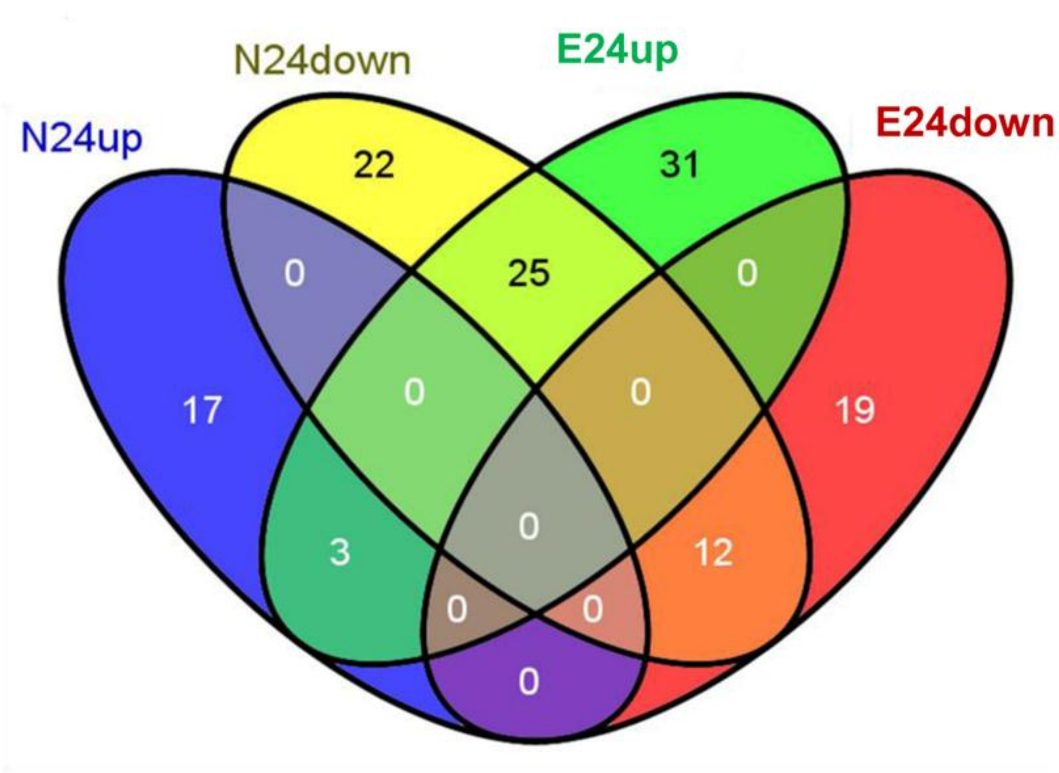

B. Venn diagram illustrating the numbers of differently up- and down-regulated miRNAs between Nan Dan Ba Di Da Hua and Earlistaple 7 in response to salt stress at 24h

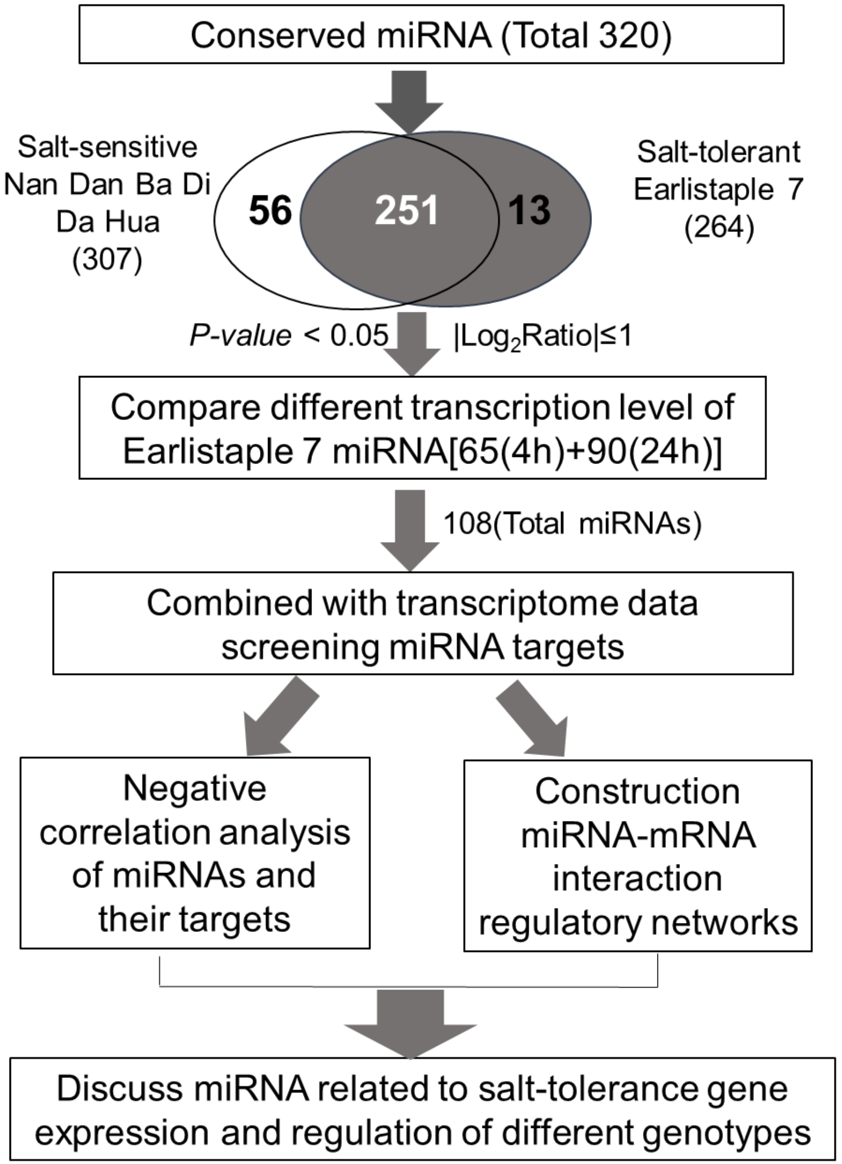

C. Flowchart analysis of the differentially expressed miRNAs and their target genes

Combined with transcriptome data screening miRNA targets

Between-Sample	miRNA	Unigene	relationship
E4/E0	64	467	761
E24/E0	87	561	1016

Chosen Negative relationships |Log_2|≤ 1 *P-value* < 0.05

miRNA-Target genes negative correlation analysis

Between-Sample	miRNA	Unigene	relationship
E4/E0	52	84	138
E24/E0	63	118	200

Eliminate no annotation

Number of the final miRNA-Target gene relationships

Between-Sample	miRNA	Unigene	relationship
E4/E0	41	44	65
E24/E0	52	73	101

D. Procedure for screening of negative miRNA-target unigene relationships related to salt-treatment response

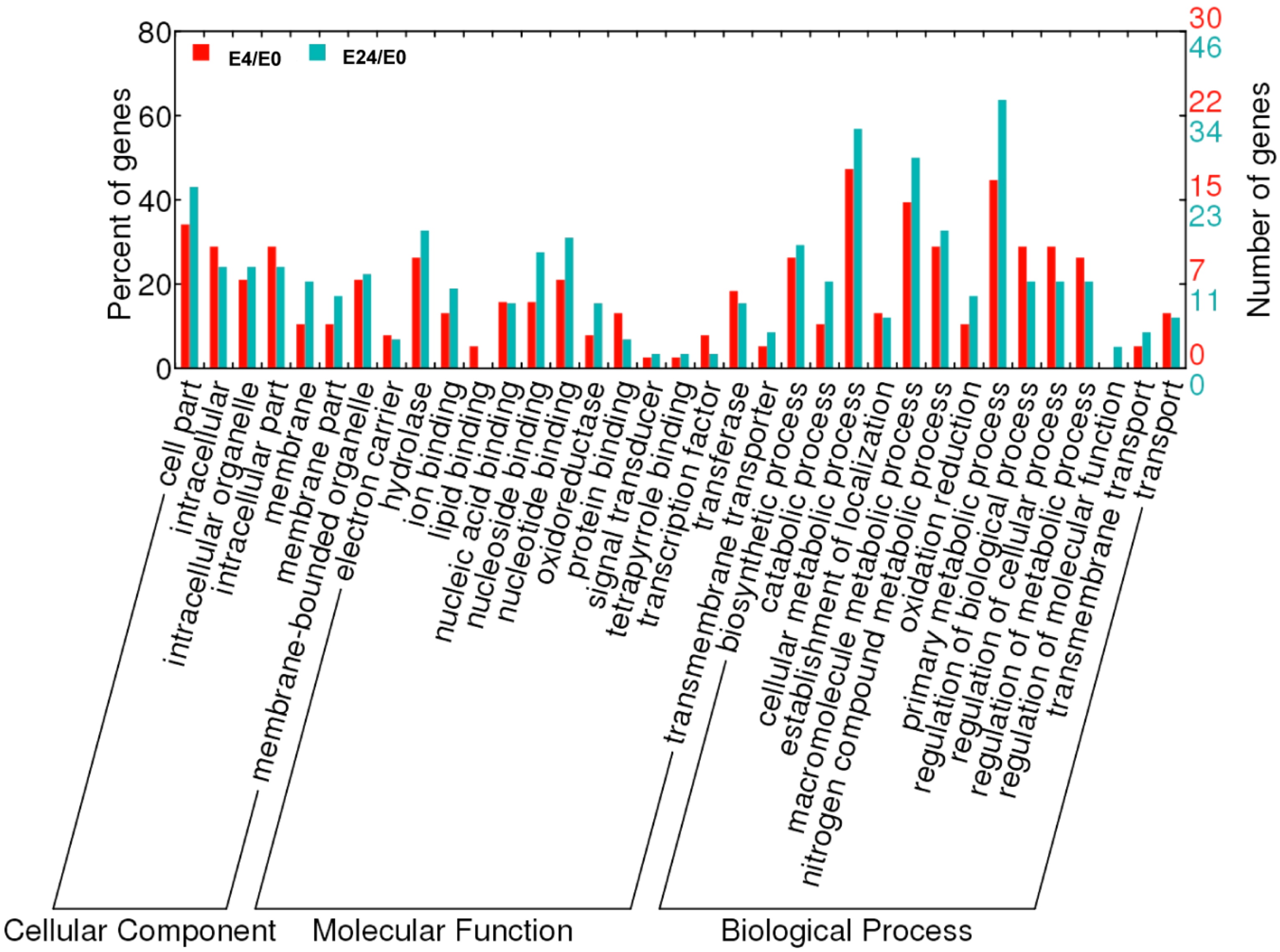

E. Gene Ontology analysis of conserved miRNA targets identified in salt-tolerant Earlistaple 7 at 4 and 24 h of salt treatment.
